# Trends in Alzheimer's Disease Research Based upon Machine Learning Analysis of PubMed Abstracts

**DOI:** 10.7150/ijbs.35743

**Published:** 2019-08-06

**Authors:** Renchu Guan, Xiaojing Wen, Yanchun Liang, Dong Xu, Baorun He, Xiaoyue Feng

**Affiliations:** 1 Key Laboratory of Symbolic Computation and Knowledge Engineering of the Ministry of Education, College of Computer Science and Technology, Jilin University, 130012, Changchun, China; 2 Zhuhai Sub Laboratory, Key Laboratory of Symbolic Computation and Knowledge Engineering of the Ministry of Education, Zhuhai College of Jilin University, 519041, Zhuhai, China; 3 Department of Electric Engineering and Computer Science, and Christopher S. Bond Life Sciences Center, University of Missouri, 65201, Columbia, USA

**Keywords:** Alzheimer's disease, Latent Dirichlet Allocation, Affinity Propagation

## Abstract

About 29.8 million people worldwide had been diagnosed with Alzheimer's disease (AD) in 2015, and the number is projected to triple by 2050. In 2018, AD was the fifth leading cause of death in Americans with 65 years of age or older, but the progress of AD drug research is very limited. It is helpful to identify the key factors and research trends of AD for guiding further more effective studies. We proposed a framework named as LDAP, which combined the **l**atent **D**irichlet **a**llocation model and **a**ffinity **p**ropagation algorithm to extract research topics from 95,876 AD-related papers published from 2007 to 2016. Trends and hotspots analyses were performed on LDAP results. We found that the focus points of AD research for the past 10 years include 15 diseases, 15 amino acids, peptides, and proteins, 9 enzymes and coenzymes, 7 hormones, 7 carbohydrates, 5 lipids, 2 organophosphonates, 18 chemicals, 11 compounds, 13 symptoms, and 20 phenomena. Our LDAP framework allowed us to trace the evolution of research trends and the most popular areas of interest (hotspots) on disease, protein, symptom, and phenomena. Meanwhile, 556 AD related-genes were identified, which are enriched in 12 KEGG pathways including the AD pathway and nitrogen metabolism pathway. Our results are freely available at https://www.keaml.cn/Alzheimer.

## Introduction

Between 2010 and 2015, the number of Alzheimer's disease (AD) cases has more than tripled, and the prevalence of AD is still increasing 1,2. 2015 report estimated that 29.8 million people had been diagnosed with AD 3,4. As a chronic neurodegenerative disease, AD is the cause of 60% to 70% of dementia cases and in 2015, the G8 nations endorsed AD as a major societal concern when it was reported as the cause of about 1.9 million deaths 5-9. Moreover, the number of global AD cases is projected to more than triple by 2050 2,10,11. In 2018, AD became the sixth leading cause of death in the United States and the fifth leading cause of death in Americans with 65 years of age or older 12. It is predicted that by 2050, the number of deaths caused by AD will account for 43% of all deaths among the elderly in the United States 4.

AD is a type of brain disease that begins primarily at the age of 65 or older, although 4% to 5% of AD cases are early-onset 13. The early symptom in AD is difficult in remembering recent events, also called short-term memory loss 6,14. Symptoms arise because part of the brain's nerve cells (neurons) that are involved in thinking, learning, and memory (cognitive function) have been damaged or destroyed^15^. As the disease advances, symptoms may include problems with language, disorientation, and some pathological signs and symptoms such as atrophy and paralysis. As an AD sufferer's physical condition declines, much of his or her basic bodily functions are gradually lost, and this will ultimately lead to death 16. Although the health situation may vary after diagnosis, the life expectancy is 3 to 9 years on average 17,18.

Many journals, conferences, patents, and associations are dedicated to the study of AD. For example, Alzheimer's Association releases an annual report, which describes the public health impact of AD, including incidence and prevalence, mortality and morbidity, use and costs of care, and the overall impact on caregivers and society 19. There are nearly 0.6 million authorized patents about AD. With the fast development of biotechnology and medicine, a huge amount of biomedical literature and data have been published. For example, until February 2019, 142,785 papers have been published on AD in PubMed. From these research, we can find the research hotspots and trends of AD.

For the curation research on AD, the whole world is struggling to find more effective ways to treat the disease, delay its onset and prevent its development. However, this progress is not satisfactory. For example, it is reported that researchers had spent more than 100 billion dollars to find effective drugs to cure AD in clinical treatments in past years. But the results were disappointed as in the failure of the antibody, solanezumab, which held the promise of improving cognition but failed trial tests 20. Alzheimer's disease is currently incurable, but treatments for symptoms are available. Although current Alzheimer's treatments cannot stop the disease progressing, they can temporarily slow the deterioration of dementia symptoms and improve the quality of life 14,21. In 2018, it is reported that more than 16 million family members and other unpaid caregivers provided approximately 18.5 billion hours of care for people with Alzheimer's or other dementia. The value of this care is close to $234 billion 15. AD is considered as one of the most financially costly diseases in developed countries 22,23.

For clinicians or biological researchers, rapid and effective acquisition of cutting-edge information on research advances from tens of thousands publications is a huge challenge 24. However, because of the complexity of the multi-disciplinary study, there is no research to summarize these findings to keep pace with the research trends of AD using machine learning and natural language processing models. To address this problem, we adopted machine learning methods to automatically summarize the trends and hotspots of AD based on PubMed abstracts. For this purpose, we collected 10 years of abstracts on AD research from PubMed. Then we proposed a framework named as LDAP which combined latent Dirichlet allocation model and the affinity propagation algorithm to extract research topics and hotspots. By incorporating the Medical Subject Headings term categories, we studied the trends includes medical entity changes in diseases, chemicals and drugs, symptoms and phenomena, and gene pathway based on the hotspots for the past 10 years. There may be other trends changed about AD, but in this study, we only focus on the changes in the areas mentioned above.

## Methods

### LDA topic model

Latent Dirichlet allocation (LDA) is a topic model algorithm based on probability proposed by Blei *et al*. in 2003 25. It is an unsupervised machine learning technique and can be used to identify potentially hidden topic information in large-scale document sets or corpora 26. LDA is a document theme generation model, which also known as a three-layer Bayesian probability model. It assumes that each word is extracted from a hidden theme behind it, and it contains three layers of words, topics, and documents 27,28. LDA assembles a collection of documents, where *D*={*d_m_*}, *m*∈{1, …, *M*}. The distribution of topic *k* over vocabulary is denoted as Φ={*ϕ^k^*}, *k*∈{1, …, *K*}, and the distribution of the *m-*th document over all *K* topics is denoted as Θ={*θ_m_*}, *m*∈{1, …, *M*}. A topic is a distribution of terms over a vocabulary. It allows each document to be described as a distribution over topics, which can be expressed as:


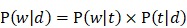
(1)

where *w* represents a word, *d* represents a document, and *t* is the topic. It can be expanded as follows:


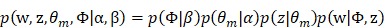
(2)

where, for document *m*, the distribution of document over topics *θ_m_* and the distribution of topics over vocabulary Φ are sampled from priors *α* and *β*, respectively. Then, the topic assignment *z* for each word is generated from *θ_m_*, and the accurate words *w* are generated according to their respective topic assignment *z* and the distribution of topics over the vocabulary Φ.

To get the topic words of each year, we used Gibbs sampling to estimate the LDA model. Gibbs sampling is a Markov chain Monte Carlo (MCMC) algorithm for obtaining a sequence of observations which are approximated from a specified multivariate probability distribution, when direct sampling is difficult.

### Affinity Propagation model

Affinity propagation (AP) is a clustering algorithm based on the concept of “message passing” between data points. AP finds "exemplars", which are members of the input set representing clusters 29. AP has been used in many fields, such as image processing 30, text clustering 31, and gene detecting 32.The description of an AP algorithm is as follows: Given a sample set *X* = {*x*_1_*, x*_2_*, ..., x_n_*}, and there is no hypothesis of inherent structure between data. Let **S** be a matrix that depicts the similarity between points, *s*(*i*, *j*) > *s*(*i, k*) if and only if the similarity between *x_i_* and *x_j_* is greater than that of *x_i_* and* x_k_*.The similarity is represented by the reciprocal of cosine similarity corresponding to (3):


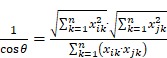
(3)

The AP algorithm alternates between two messages passing steps to update two matrices:

(a) The responsibility matrix **R**: *r*(*i*, *k*) describes the extent to which sample *k* is suitable for the clustering center of sample *i*, and it represents the messages from *i* to *k*.

(b) The availability matrix **A**: *a*(*i*, *k*) describes the selection sample *i* choosing sample *k* as the degree of suitability for the cluster center, and it represents the messages from *k* to *i*.

The matrix **R** will be constantly updated according to (4).


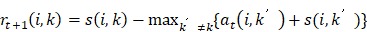
(4)

The matrix **A** will be continually updated according to (5) and (6).


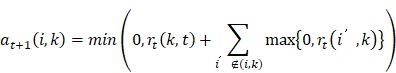



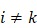
(5)


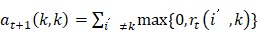
(6)

After copious iterations, the final clustering result can be obtained when the two matrices converge. To avoid numerical oscillations, a damping factor *λ*∈(0,1) is introduced when updating the two matrices, as described in (7) and (8):


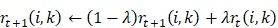
(7)


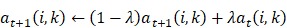
(8)

### Proposed framework

**Figure 1:**
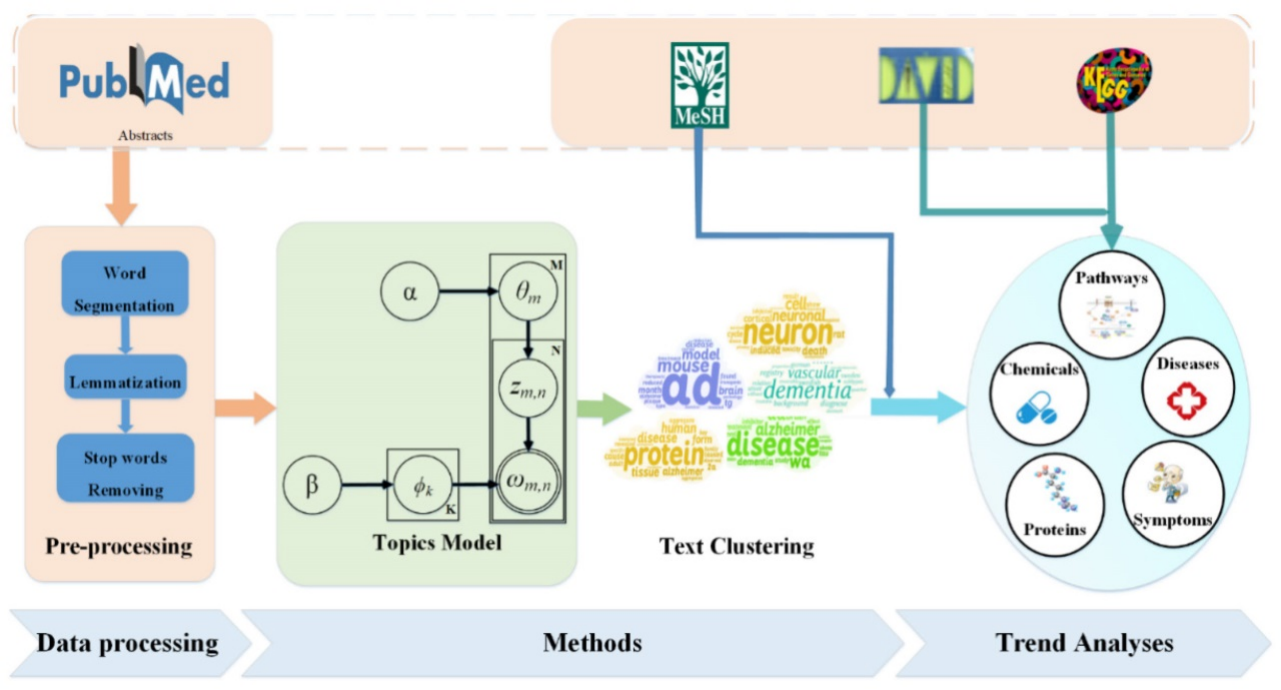
** Proposed framework**. This framework consists of data processing, methods, and trends analyses. Data downloaded from PubMed was pre-processed and fed into an LDA model. AP algorithm was used to cluster the topics generated from LDA. Trend analyses were done on these topic words.

This paper presents an LDAP framework, which combines LDA model and AP algorithm to extract research topics. Our proposed framework is depicted in Figure 1. It consists of data processing, methods, and trends analyses. The data from PubMed are downloaded and pre-processed with operations of word segmentation, lemmatization, and stop words removal techniques. After data acquisition and preprocessing, the data is fed to the topics model LDA. The evaluation index, perplexity, in the LDA model can cause some variation in the number of topics. When the number is too small, the model may not reach convergence and achieve the optimal result; meanwhile, the model will lose the ability of capturing topic-diversity and this can cause highly used words like *ad, disease, alzheimer*, and *dementia* to get high scores in each topic. Therefore, we generated topics using LDA and then clustered them with AP. With the introduction of MeSH, David, and KEGG, the results can be represented from different aspects, such as proteins, diseases, and pathways.

## Experiments and Results

### Data set and preprocessing

Because an abstract can provide a concise and accurate description of the important content of a paper, necessary information can be obtained from abstracts without reading the full text. We took “Alzheimer's disease” as the entry term to search papers in PubMed and obtained 95,876 papers from 2007 to 2016. Each file is a semi-structured XML document and contains various tags, such as <title>, <abstract>, <pmid>, etc. We extracted the content in <abstract> and <pmid> fields from the raw XML files. PMID is the unique ID for a paper in PubMed. After extraction, each abstract was stored in a corresponding file.

**Figure 2:**
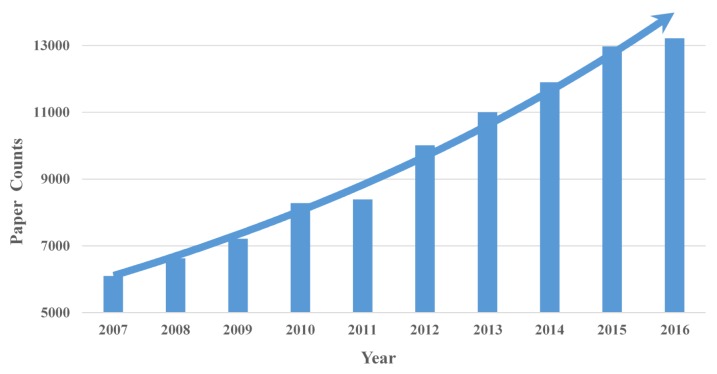
** Paper counts on AD from 2007 to 2016.** The number of papers on AD increased over time, and the total number of ten years is 95,876.

We obtained statistics in which the number of papers on AD increased over time. Figure 2 shows that with the aging of society and understanding of AD, more and more research is devoted to the study of AD.

Because the original data contains irrelevant and noisy information that affected the correctness of the final experimental results, a pre-processing operation was necessary to assure the accuracy of the dataset. In our work, the natural language processing techniques such as word segmentation, lemmatization, and stop words removal were applied to the raw data.

### Parameters

In our experiment, for LDA model, we set the topic number *t*=200, hyper-parameters *α*=0.25, *β*=0.01, and the iteration *i*=400 by perplexity evaluation. In the AP algorithm, we set the damping factor to 0.95 after several adjustments.

### Topic results

**Figure 3:**
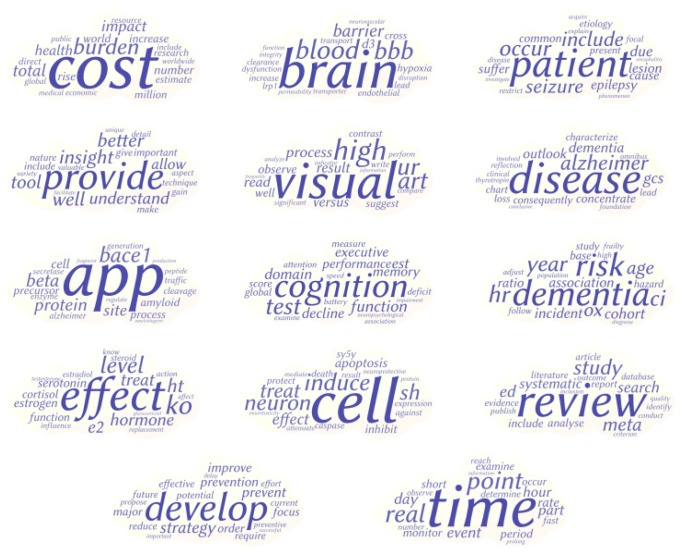
** AD research topics of 2016.** LDAP results contain 14 clusters and each cluster is represented by 20 words. Word cloud displays different size words. The more weight given a word, the more prominent it is in AD terminology.

Our LDAP framework adopts the LDA model to process the annual data, and 200 topics are obtained for each year. Then the AP algorithm is employed to cluster the LDA results. We used word clouds to visualize the LDAP results. Word cloud displays different size words where the higher the word weight, the bigger the word. Taking the results of 2016 as an example, as shown in Figure 3, the LDAP framework generated 14 clusters, and the central topic words are summarized as “patient”, “cost”, “develop”, “time”, “review”, “cell”, “effect”, “provide”, “app”, “visual”, “brain”, “cognition”, “dementia”, and “disease”.

In Figure 3, *cost* is the central word of the first cluster, and the other obvious words such as *burden*, *health*, and *million* are also associated with cost. The caring for persons with AD has caught great attention. AD is considered as one of the most financially costly diseases in developed countries. Moreover, *App* (Amyloid Precursor Protein) is another central word that can present the whole cluster of protein topics, including beta amyloid protein and bace1 which are directly associated with AD. More research topics from 2007 to 2015 are shown in Supplemental Figure S1-S9.

The topic centers (most used AD terminology, i.e., words associated with hotspots) are presented by years and listed in Table 1. The word after each forward slash (/) is the central word of the biomedical aspect implied in the result, and it also appears in the results at the same time. Some topic-center words such as brain, dementia, cognition, and protein, appear in most of the years and are well-known AD research hotspots. On the contrary, the topic center word, *education*, appeared only once in 2015. We found a hotspot on the link between education and dementia in 2015 33.

There are 142 clusters for all ten years' data and 20 words in each central topic. We summed up all the words in the central topics and obtained 1,988 unique words. Then we introduced the Mesh terms to classify the categories of these words, and obtained 75 categories in all, which are: 15 diseases, 15 kinds of amino acids, peptides, and proteins, 9 enzymes and coenzymes, 7 hormones, 7 carbohydrates, 5 lipids, 2 organophosphonates, 18 chemicals, 11 types of compounds, 13 different symptoms, 20 different phenomena, and other categories. In our experiments, we focused our research on 11 categories and recorded the trends and hotspots that the center topics words represent. In the following section, we analyzed trends and hotspots on terminology pertaining to diseases, proteins, symptoms and phenomena, and gene pathways.

### Disease hotspots

**Figure 4:**
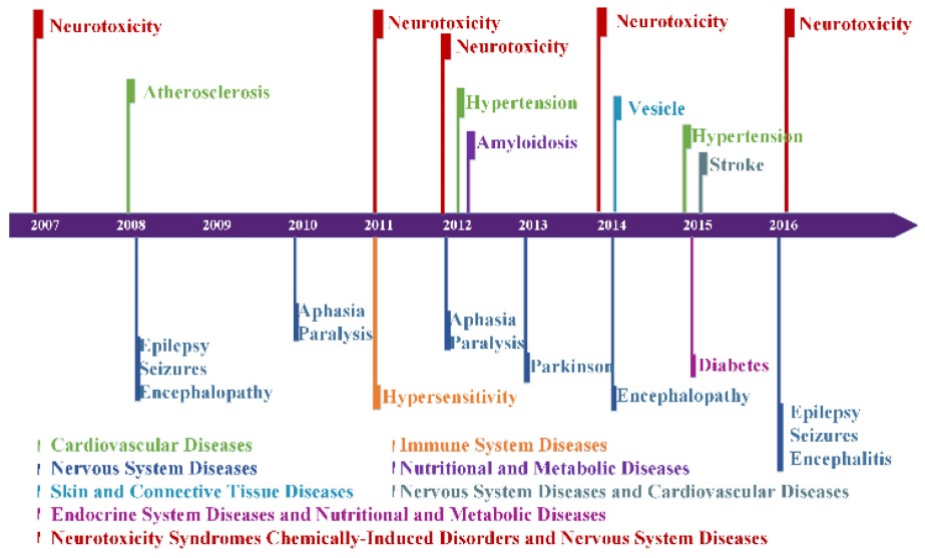
** Disease associations with AD in each year.** Different colors represent different categories of diseases. *Neurotoxicity*, *Stroke*, and *Diabetes* belong to two categories of diseases at the same time.

Figure 4 illustrates 15 diseases or disorders related to AD. They are amyloidosis, atherosclerosis, hypertension, stroke, diabetes, hypersensitivity, aphasia, encephalitis, encephalopathy, epilepsy, paralysis, Parkinson's, seizures, neurotoxicity and vesicle, which can be categorized into six types of diseases and one type of disorder. For example, amyloidosis belongs to nutritional and metabolic disease, aphasia and Parkinson's are nervous system diseases.

Figure 4 shows that neurotoxicity had appeared 5 times in ten years. Neurotoxicity is a form of toxicity that adversely affects the nervous system and was reported as having a strong relation with Alzheimer's disease. Besides neurotoxicity, some diseases appeared in the same year; for example, aphasia and paralysis appeared in 2010 and 2012, and epilepsy, seizures, and encephalopathy or encephalitis appeared in 2008 and 2016. Aphasia and paralysis are usually associated with symptoms after a stroke. Epilepsy and encephalopathy can damage the nerves of brain. This suggests that these diseases are not only related to Alzheimer's disease, but also have relations among themselves.

**Figure 5:**
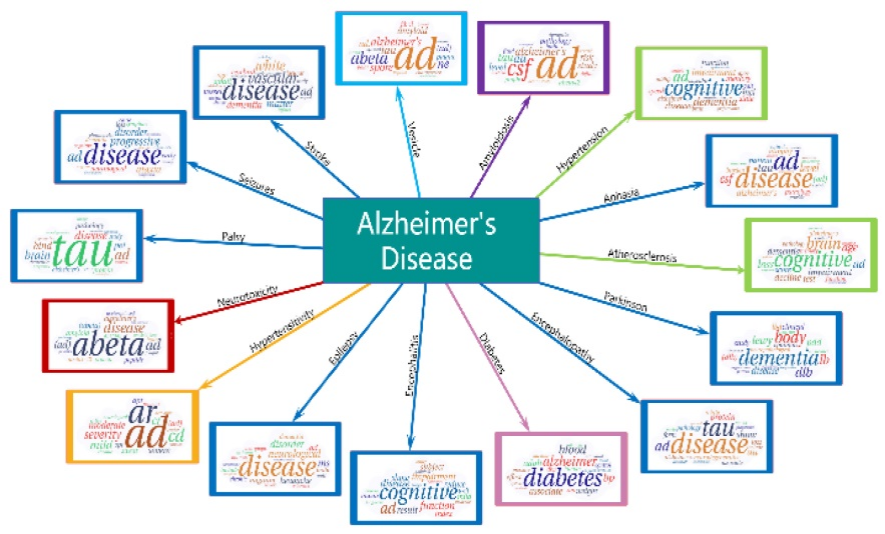
** AD emerges in 15 diseases hotspots.** The arrow indicates different diseases and the word clouds are the hotspots of them.

For the 15 diseases identified from the hotspots on Alzheimer's disease, we downloaded the abstracts for each disease from 2007 to 2016 to double check the relation between AD and these diseases. We used the same model to process these 15 diseases to find whether AD appeared in the results. The result is shown in Figure 5. It shows that Alzheimer's disease also appeared in the hotspots of these 15 diseases. Besides, the association between alzheimer's disease and diabetes can be found in the research 34, 35. This finding may be helpful in the early diagnosis of AD and these diseases.

### Chemicals and drugs hotspots

**Figure 6:**
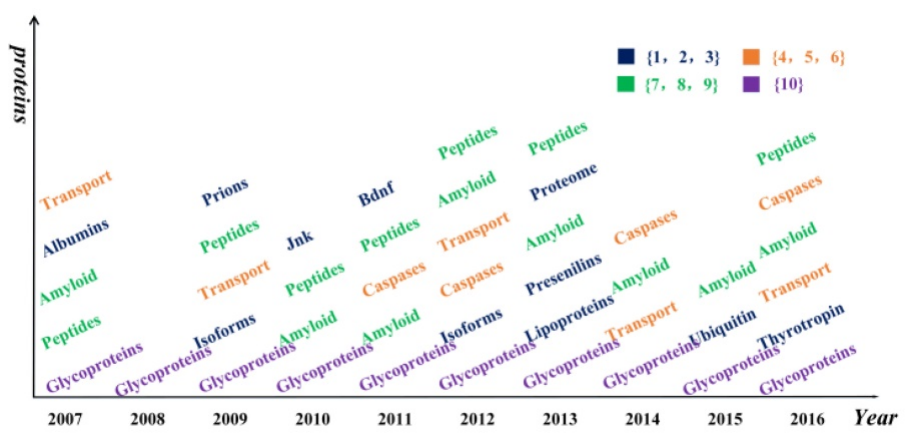
** Hotspot words on protein in each year.** Different colors represent the frequencies of different kinds of proteins. The numbers in brace are the frequencies of proteins appeared.

Besides diseases, other entities related to Alzheimer's disease are also found in the results, such as enzymes, hormones, and lipids. The distribution of proteins in each year is shown in Figure 6. The distribution of other categories in each year is shown in Supplemental Table S1-S3.

Glycoproteins appeared in all years, and amyloid appeared in 8 years from 2007 to 2016, except for 2008 and 2009. It is well known that amyloid and glycoprotein are important to AD 36, 37. Apart from these words, some chemicals and compounds were found in the results, such as analgesics, estrogens, glucocorticoids, statin, and protease inhibitors, as listed in Table 2. We found the study of chemicals did not appear in 2015. And the study of the organic chemical was concentrated from 2008 to 2010.

### Symptoms and phenomena hotspots

**Figure 7:**
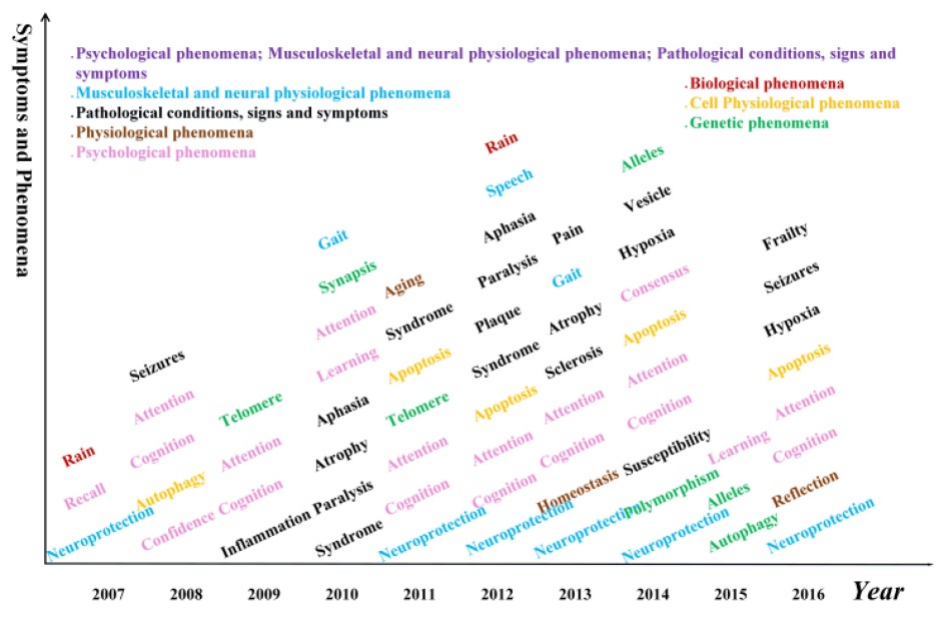
** Hotspot words on symptom and phenomena in each year.** Different colors represent different categories of symptoms or phenomena.

It is well known that AD patients have difficulty in remembering recent events and have problems with language. However, other symptoms and phenomena are also common, such as aphasia and telomere. The complete list of symptoms and phenomena from our data is shown in Figure 7.

Apart from the decline of memory, the cognition, attention, memory recall, learning and speech abilities of AD patients are also declined. In addition, inflammation, neuroprotection, telomere, syndrome, synapsis also appeared, which could potentially suggest a composite screening or early warning of Alzheimer's disease based on these symptoms.

### Gene pathway hotspots

We identified all the genes appeared in the topics, and 556 genes were found in total. To analyze these genes, we uploaded these genes to the David database 38 to find the enriched pathways of these genes.

As a result, 12 pathways were achieved; two of them are the Alzheimer's disease pathway and the nitrogen metabolism pathway. In the AD's pathway, 12 genes were taken from our results, including the ADAM metallopeptidase domain 10 (ADAM10), LDL receptor related protein 1 (LRP1), amyloid beta precursor protein (APP), apolipoprotein E (APOE), beta-secretase 1 (BACE1), cyclin dependent kinase 5 (CDK5), microtubule associated protein tau (MAPT), insulin degrading enzyme (IDE), presenilin 1 (PSEN1), presenilin 2 (PSEN2), synuclein alpha (SNCA) and tumor necrosis factor (TNF). The P-value of the pathway is 4.20E-07, and the false discovery rate (FDR) is 5.08E-04 according to our uploaded genes. Both P-value and FDR are less than 0.01, indicating that the pathway of our uploaded genes is statistically significant.

**Figure 8:**
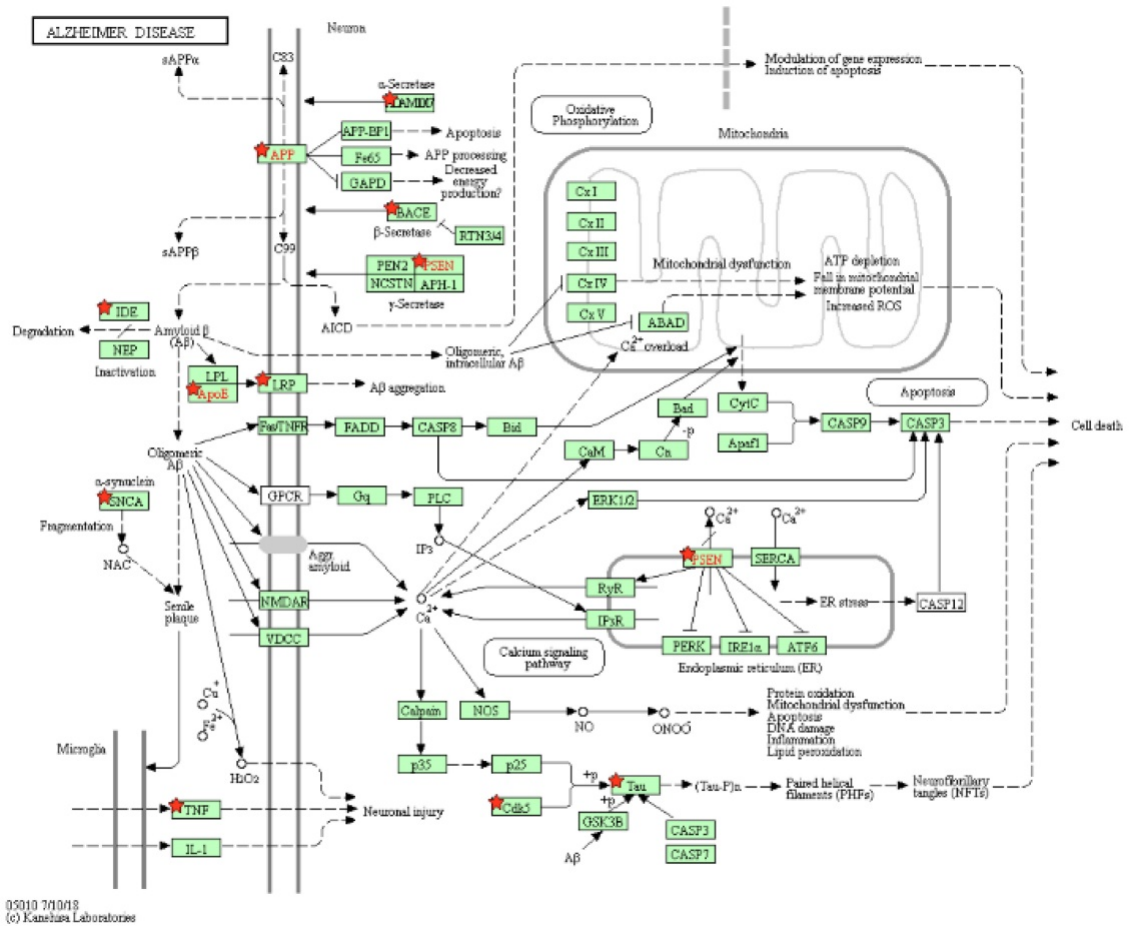
** Pathway of Alzheimer's disease.** Our uploaded genes are marked by red stars. The red genes are labeled by KEGG, and they are in our results.

The pathway of Alzheimer's disease is shown in Figure 8, and the genes in our results are marked by red stars to distinguish them with other genes. The original source of this pathway is from the KEGG website 39, 40. The other 11 pathways are shown in Supplemental Table S4 and Figure S10-S20.

### AD research trends

**Figure 9:**
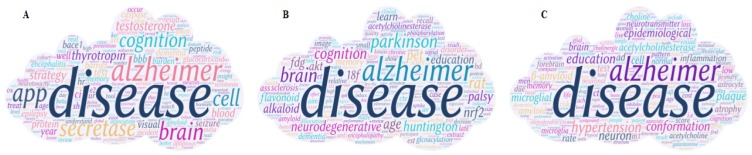
** Key topics of AD from 2016 to 2018.** (A) 2016 key topics. (B) 2017 key topics. (C) 2018 key topics. Different size of words displays the word's weight. The higher the word weight is, the bigger the word is.

We analyzed the trends and hotspots on center topics and three other aspects based on center topic words. AD research continues to evolve year after year, and there are some latent tendencies in the past years. To capture these tendencies, we merged all the central topics of the whole year into a word cloud. Taking 2016 as an example, the visualization of the result is shown in Figure 9A. *Alzheimer*, *disease*, *time*, *brain*, *app*, *cost*, and *cell* are the most noticeable words represented the research hotspots in 2016. The other figures from 2007 to 2015 are shown in the supplemental material as S21-S29.

Combined with the results of the 10-year (from 2007 to 2016) word clouds, we can infer that (1) phenomena, amino acids, peptides, and proteins appeared in each year and the disease category appeared in 9 years (except 2009). These categories are considered as long-term research hotspots and directly related to AD. Therefore, we can predict that these categories would be the hotspots topics in 2017 and 2018. To validate the prediction, we generated AD-research hotspots in 2017 and 2018 using LDAP and achieved the validation. We found these three categories in Figure 9B and 9C, where disease category appeared on the upper part of the word clouds, as *Parkinson, Huntington, palsy, Neurodegenerative Diseases, encephalopathy, and sclerosis* appear in 2017 and *hypertension* appears in 2018. (2) From Figure 4, we find that hypertension occurs in 2012 and 2015 with a time interval of 3 three years, therefore, we predict that it will appear in 2018. In the result of 2018 (Figure 9C), we found it in pink under the “disease” topic.

**Figure 10:**
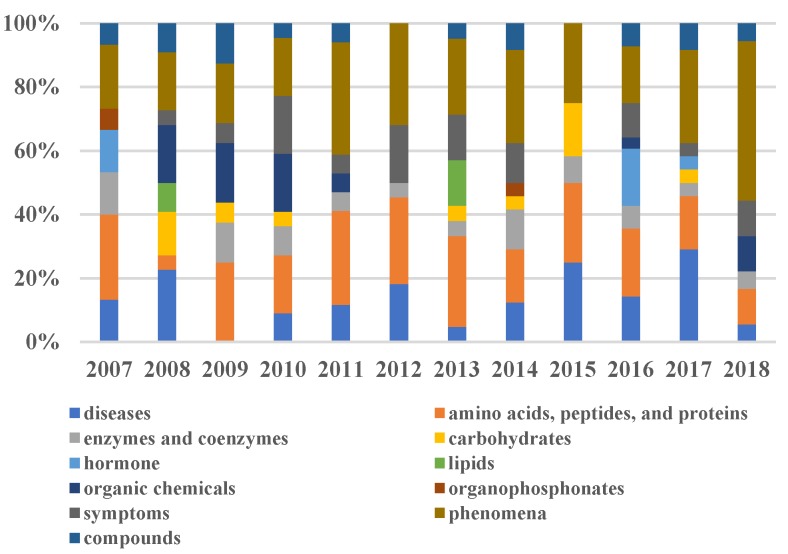
** Proportion of each category about the hotspots from 2007 to 2018.** Different colors represent different categories. The height of each color indicates the proportion of that category.

In addition, the proportion of MeSH category about the hotspots from 2007 to 2018 is shown in Figure 9. Different colors represent the proportion of different types of research in a year. In Figure 10, the hormone category appeared in 2007 and 2016. In 2016, the proportion of hormones was twice that of 2007. It implies that the research on hormone has made new discoveries for AD around 2016. We found clues from the researches on whether the growth hormone can be contaminated by amyloid-β seeds as well as by prions, which were published in *Nature* in September 2015 and November 2016 41, 42. Therefore, we also find the hormone category in 2017. These evidences certified our research trends results.

## Conclusions

To reveal the hotspots and trends of AD, we proposed a novel LDAP framework which combines topics model and AP algorithm. From about 100,000 AD papers, we found the spotlights of AD research including 556 genes, 15 diseases, 15 amino acids, peptides, and proteins, 19 chemicals and compounds, 33 symptoms and phenomena, and 12 related pathways. We studied the research hotspots via a visualization method to find the regularities, and reveal the research hotspots on AD research. It should be noted that there is no unique criteria to explain the trends of AD. In this paper, we mainly use the information of PubMed abstracts through machine learning models. This may have some bias for completely understanding the trends of AD. However, the discovery of the research trends and hotspots evolution on AD are supposed to provide some guidance for further research and might to useful to drug study. For example, a drug used to treat a disease associated with the 556 AD related genes may treat or alleviate some of the symptoms of AD. In addition, the 15 amino acids, peptides, and proteins, 9 enzymes and coenzymes, 7 hormones, 7 carbohydrates, 5 lipids, 2 organophosphonates, 18 chemicals, 11 compounds can be made into corresponding inhibitors, which may be used to treat AD. These results are required further experimental verification. In addition to the research manuscripts, patents about AD also can provide the hotspots from different views, especially for the drug development. We will introduce this data in future research.

## Supplementary Material

Supplementary figures and tables.

## Figures and Tables

**Table 1:** Topic centers of each year

Years	Topic Centers
2007	app; brain; cognition; disease; neuron; treat; dementia; affect; bind; study; effect; problem; study/gender; post; test
2008	protein; cognition; metabolic/disease; human/brain; cost; inhibit; molecule; mechanism; measure; datum; patient; rat; association; study; neurological
2009	microglia; protein; cognition; disease; inhibit; diagnose; method; study; population; provide; domain; significant; region; increase
2010	rat/memory; signal/protein; image/brain; increase; progressive/dementia; synaptic/neuron; diagnose; fibril; progressive; specific; concentration; term; therapy; measure
2011	cell; regulate/brain; cognition; disease; compound/inhibit; clinic; dementia; structure; affect; identify; case; cross; time
2012	amyloid; brain; test/cognition; disease; progressive/syndrome; mechanism; age; method; review; increase; risk; induce; month
2013	app; protein; function/cognition; metabolic; disease; dementia; identify; cortical; effect; research; volume; patient; study; develop; age; show
2014	cell; secretase; function/cognition; injury/brain; disease; evidence; dementia; year; gene; increase; predict; protein; study; control
2015	pet/amyloid; protein; rat/memory; dementia; hypertension; research; study; daily; treat; diagnose; education; progress; disease
2016	cell; app; cognition; patient; brain; cost; develop; time; review; effect; provide; visual; dementia; disease

**Table 2:** Occurrence of chemicals and compounds

Word	Categories Appearing in Each Year	
	Categories	Year
donepezil	Heterocyclic compounds; Polycyclic compounds; Organic chemicals	2009
galantamine	Heterocyclic compounds	2009
stemazole	Heterocyclic compounds; Organic chemicals	2011
rifampicin	Heterocyclic compounds; Polycyclic compounds	2014
serotonin	Heterocyclic compounds; Organic chemicals	2010, 2016
progesterone	Polycyclic compounds	2007
matrix	Polycyclic compounds	2008
oxysterols	Polycyclic compounds	2008
testosterone	Polycyclic compounds	2016
estradiol	Polycyclic compounds	2016
cholesterol	Polycyclic compounds	2013
carnitine	Organic chemicals	2008
ceramides	Organic chemicals	2008
isoflurane	Organic chemicals	2008
lactate	Organic chemicals	2008
acetylcholine	Organic chemicals	2009
ginsenosides	Organic chemicals	2009
choline	Organic chemicals	2010
inositol	Organic chemicals	2010
sulforaphane	Organic chemicals	2010
