# Supplementary material for: Trends in Alzheimer's Disease Research Based upon Machine Learning Analysis of PubMed Abstracts
Source: Int J Biol Sci. 2019 Aug 6;15(10):2065–74. doi: 10.7150/ijbs.35743 (PMC6775293; doi:10.7150/ijbs.35743)
Supplement: Supplementary file 1 — Supplementary figures and tables. [file ijbsv15p2065s1.pdf]

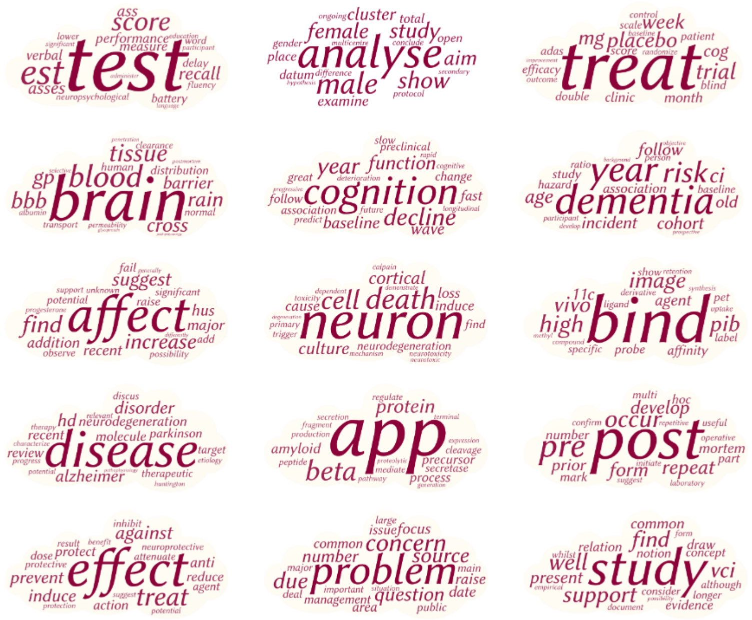

Figure S1. AD research topics of 2007. LDAP results contain 15 clusters and each cluster is represented by 20 words. Word cloud displays different size words. The more weight given a word, the more prominent it is in AD terminology.

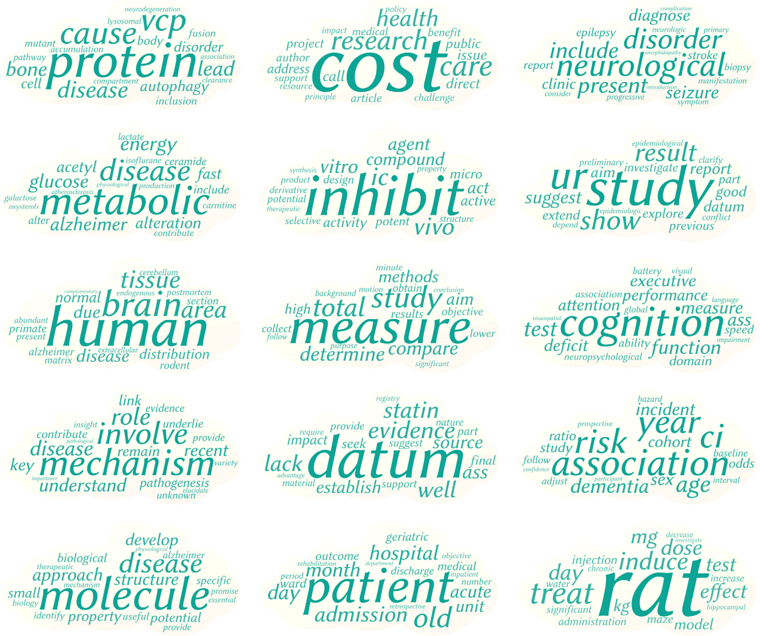

Figure S2. AD research topics of 2008. LDAP results contain 15 clusters and each cluster is represented by 20 words. Word cloud displays different size words. The more weight given a word, the more prominent it is in AD terminology.

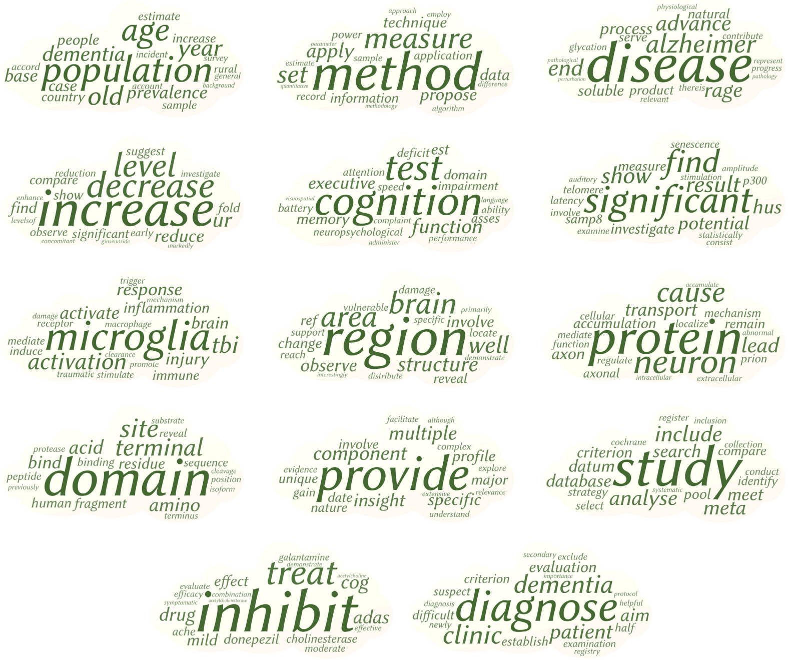

Figure S3. AD research topics of 2009. LDAP results contain 14 clusters and each cluster is represented by 20 words. Word cloud displays different size words. The more weight given a word, the more prominent it is in AD terminology.

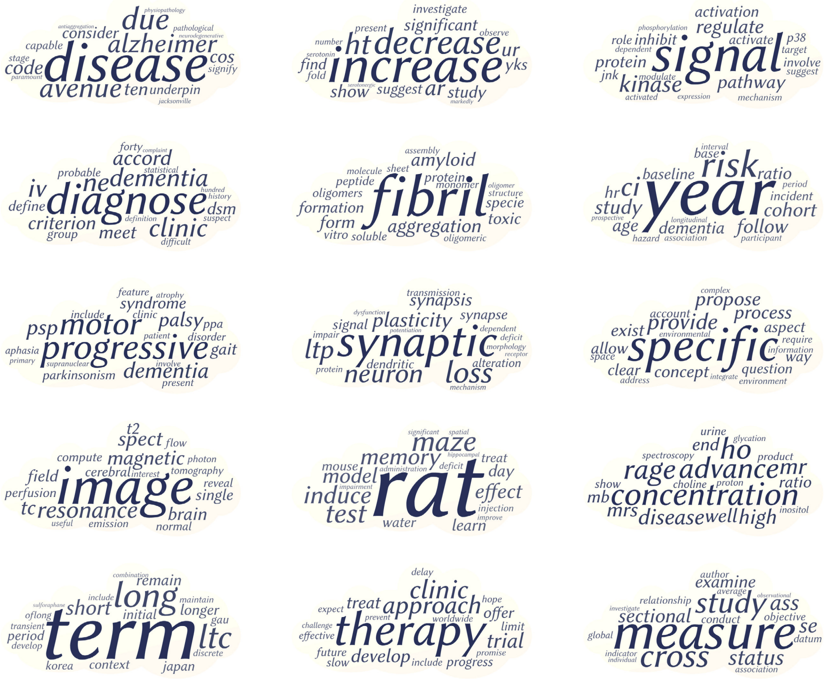

Figure S4. AD research topics of 2010. LDAP results contain 15 clusters and each cluster is represented by 20 words. Word cloud displays different size words. The more weight given a word, the more prominent it is in AD terminology.

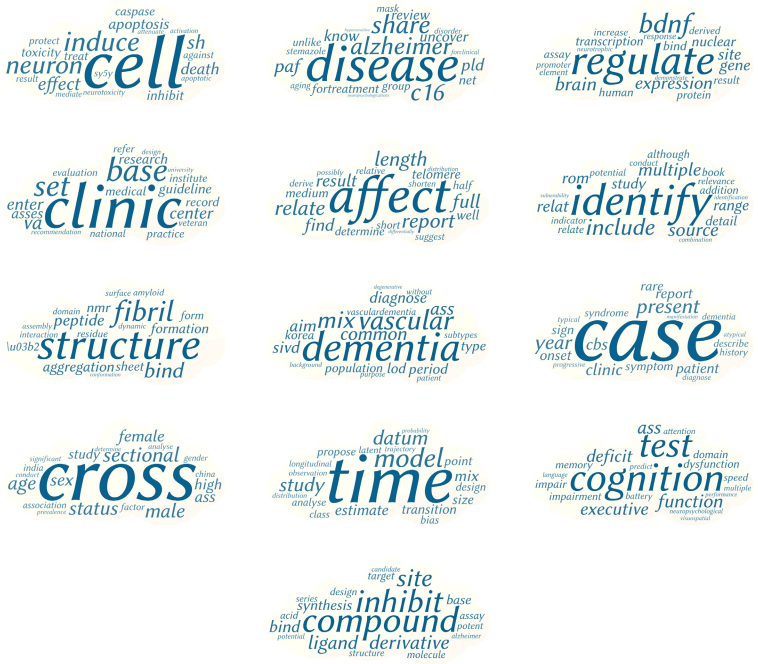

Figure S5. AD research topics of 2011. LDAP results contain 13 clusters and each cluster is represented by 20 words. Word cloud displays different size words. The more weight given a word, the more prominent it is in AD terminology.

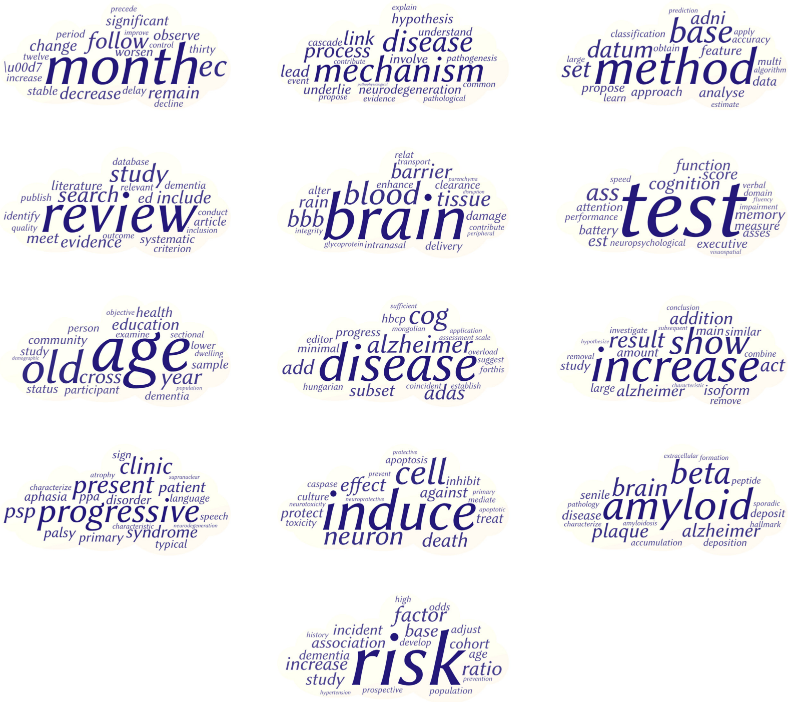

Figure S6. AD research topics of 2012. LDAP results contain 13 clusters and each cluster is represented by 20 words. Word cloud displays different size words. The more weight given a word, the more prominent it is in AD terminology.

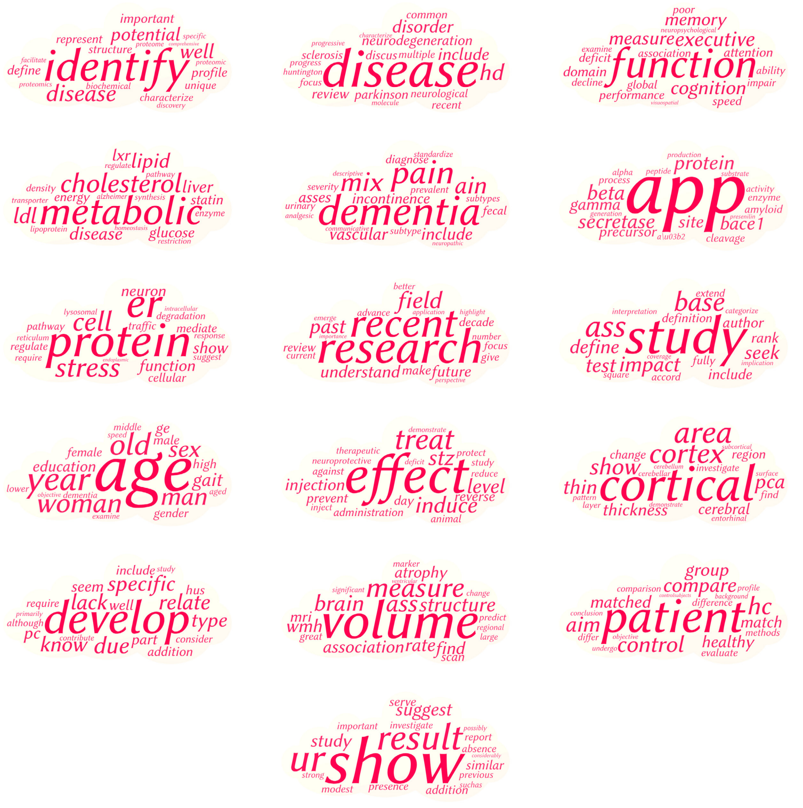

Figure S7. AD research topics of 2013. LDAP results contain 16 clusters and each cluster is represented by 20 words. Word cloud displays different size words. The more weight given a word, the more prominent it is in AD terminology.

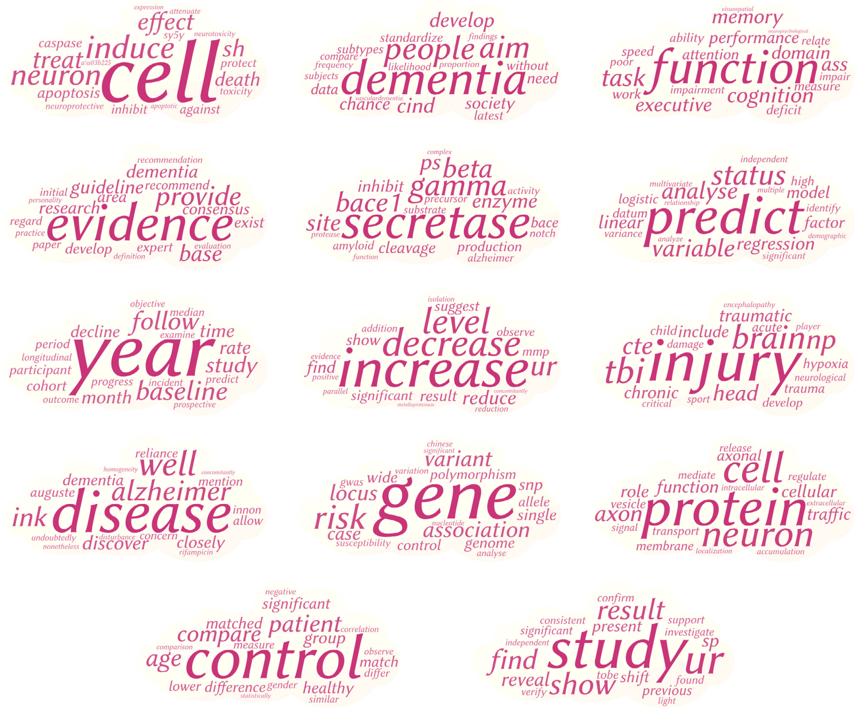

Figure S8. AD research topics of 2014. LDAP results contain 14 clusters and each cluster is represented by 20 words. Word cloud displays different size words. The more weight given a word, the more prominent it is in AD terminology.

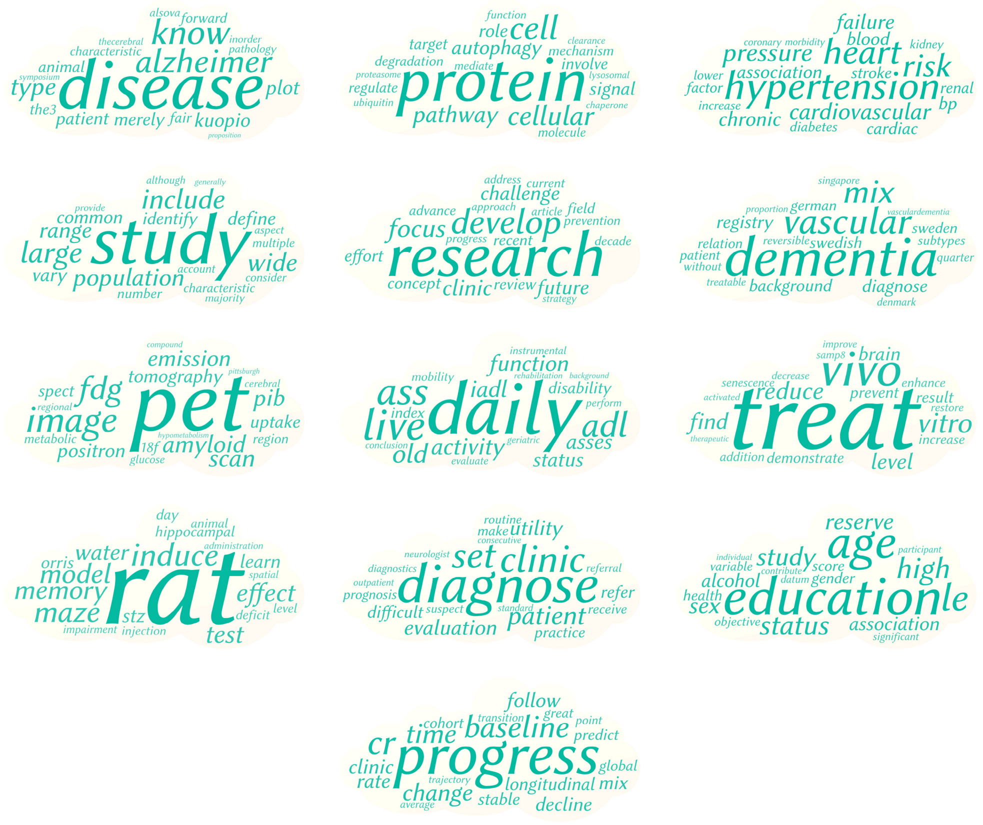

Figure S9. AD research topics of 2015. LDAP results contain 13 clusters and each cluster is represented by 20 words. Word cloud displays different size words. The more weight given a word, the more prominent it is in AD terminology.

Table S1. Occurrence of Enzymes and Coenzymes

Word	Year
(amyloid precursor protein) secretases	2007, 2013, 2014, 2016
acetylcholinesterase	2009
calpain	2007
caspases	2011, 2012, 2014, 2016
cholinesterases	2009
jnk kinase(map kinase kinase 4)	2010
kinase	2010
metalloproteases	2014
proteasome	2015

Table S2. Occurrence of Hormones

Word	Year
(hydrocortisone) cortisol	2016
estradiol	2016
glucocorticoids	2016
progesterone	2007
testosterone	2016
thyrotropin	2016
progesterone	2007

Table S3. Occurrence of Carbohydrates, Lipids, and Organophosphonates

Word	Categories Appeared In Each Year	
	Categories	Year
ceramides	carbohydrates	2008
fdg(fluorodeoxyglucose f18)	carbohydrates	2015
galactose	carbohydrates	2008
ginsenosides	carbohydrates	2009
glucose	carbohydrates	2008, 2013, 2015
inositol	carbohydrates	2010
nucleotides	carbohydrates	2014
ceramides	lipids	2008
cholesterol	lipids	2013
lipid	lipids	2013
lipoproteins	lipids	2013
oxysterols	lipids	2008
methyl	organophosphonates	2007
mmp	organophosphonates	2014

Table S4. Details of Pathway

Term	Count	PValue	FDR
bta05010:Alzheimer's disease	12	4.20E-07	5.08E-04
bta01230:Biosynthesis of amino acids	6	4.99E-04	6.03E-01
bta00910:Nitrogen metabolism	3	9.878E-03	11.33
bta05144:Malaria	4	1.17E-02	13.33
bta05014:Amyotrophic lateral sclerosis (ALS)	4	1.23E-02	13.97
bta01130:Biosynthesis of antibiotics	6	3.69E-02	36.62
bta00270:Cysteine and methionine metabolism	3	4.53E-02	42.96
bta00260:Glycine, serine and threonine metabolism	3	5.19E-02	47.58
bta04060:Cytokine-cytokine receptor interaction	6	6.12E-02	53.75
bta04330:Notch signaling pathway	3	6.62E-02	56.36
bta05030:Cocaine addiction	3	7.1E-02	59.11
bta01200:Carbon metabolism	4	7.40E-02	60.61

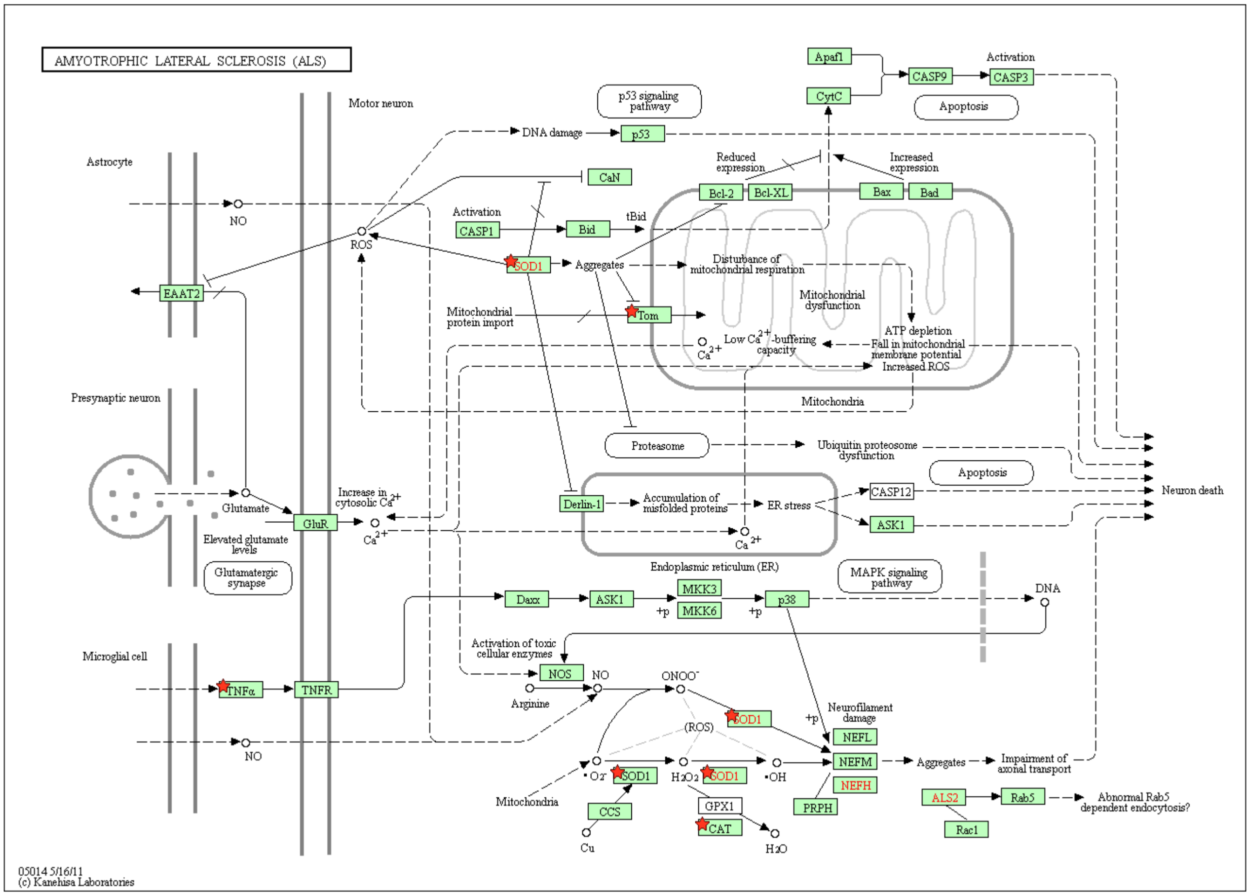

Figure S10. Amyotrophic lateral sclerosis (ALS) pathway. Our uploaded genes are marked by red stars. The red genes are labeled by KEGG, and they are in our results.

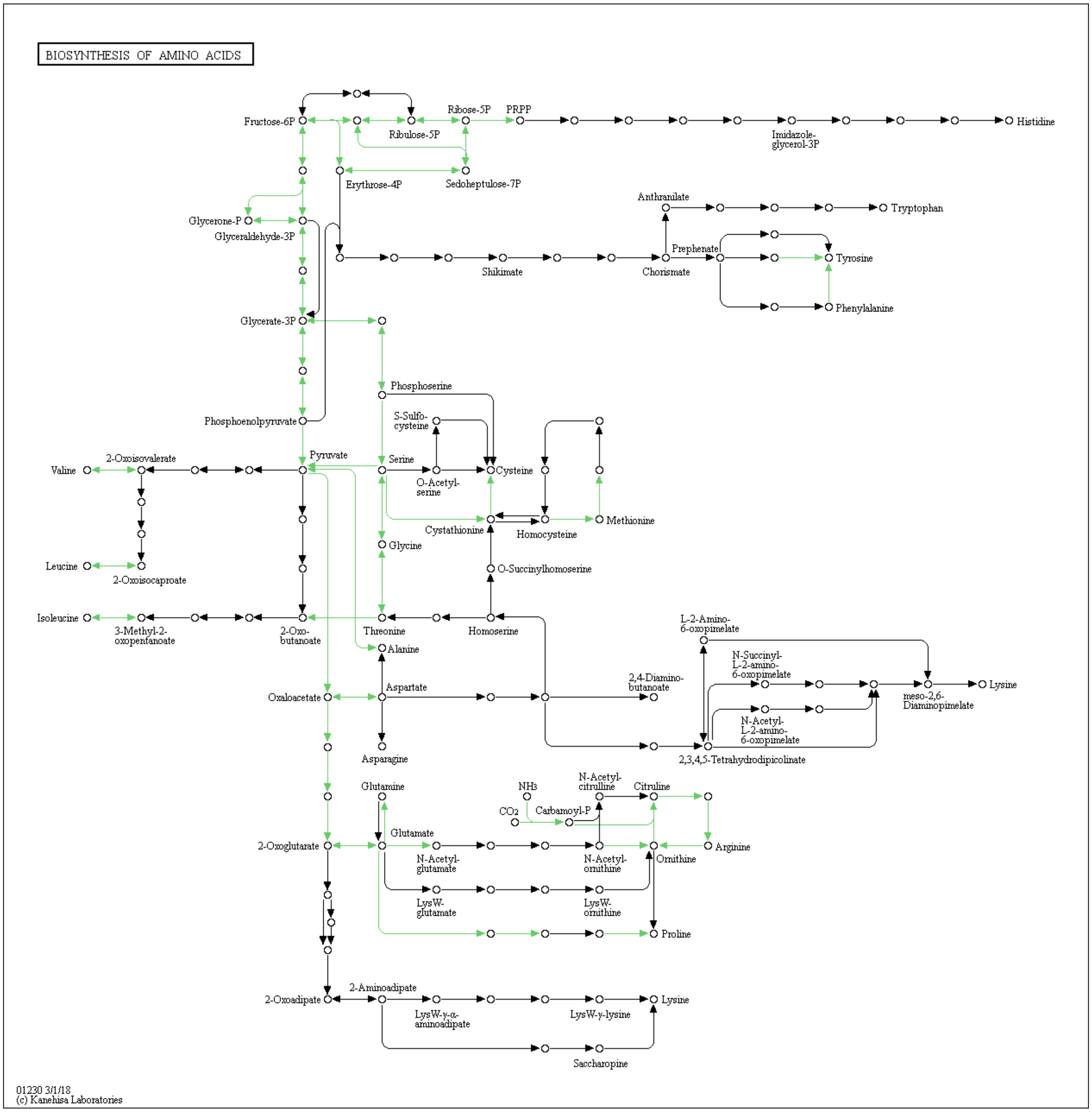

Figure S11. Biosynthesis of amino acids pathway. Our uploaded genes in this pathway are in the list of gene, but they are not labeled in the map, and they are argininosuccinate lyase (ASL), citrate synthase (CS), cystathionine-beta-synthase (CBS), pyruvate carboxylase (PC), serine dehydratase (SDS), tyrosine aminotransferase (TAT).

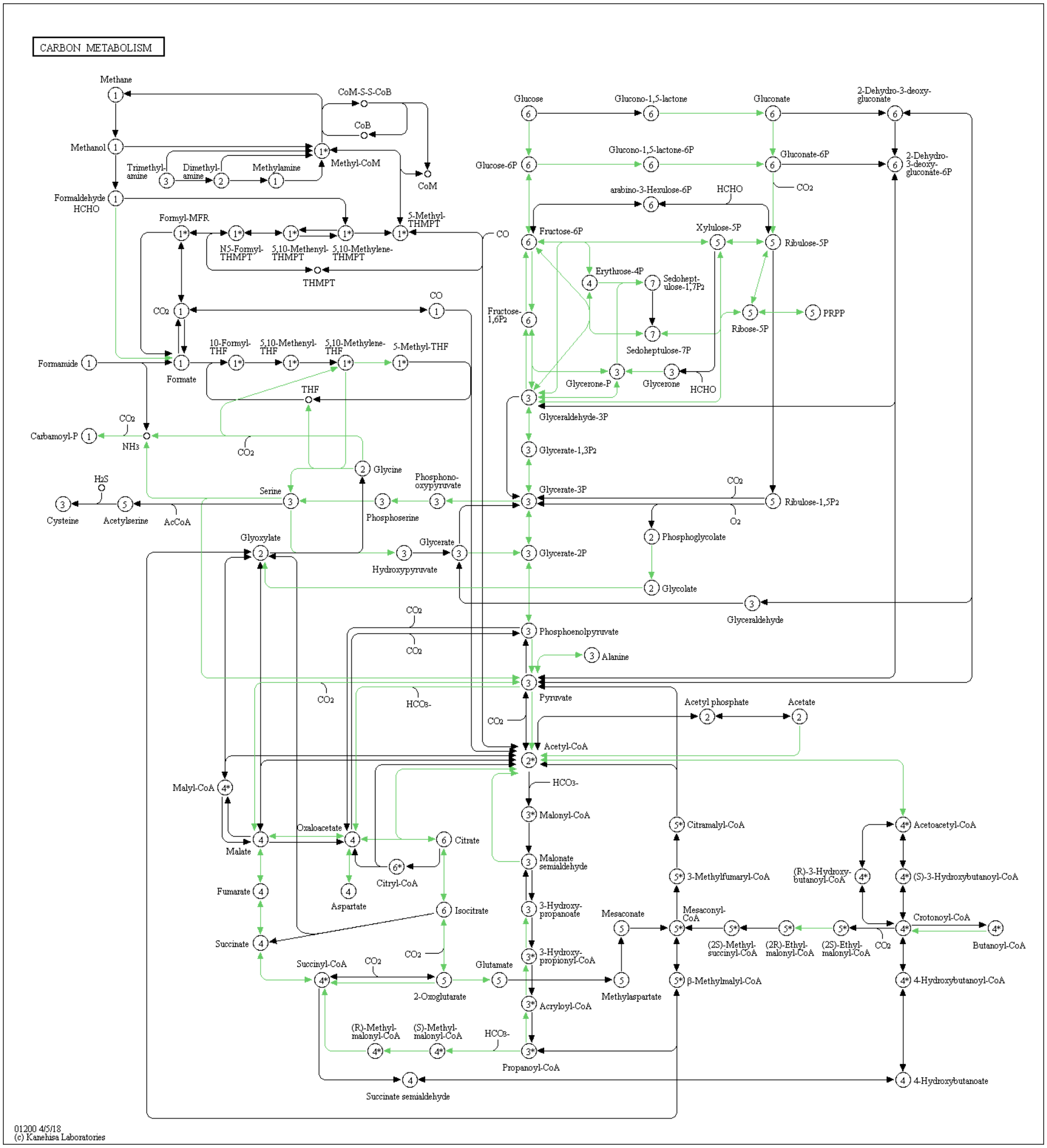

Figure S12. Carbon metabolism pathway. Our uploaded genes in this pathway are in the list of gene, but they are not labeled in the map, and they are catalase (CAT), citrate synthase (CS), pyruvate carboxylase (PC), serine dehydratase (SDS).

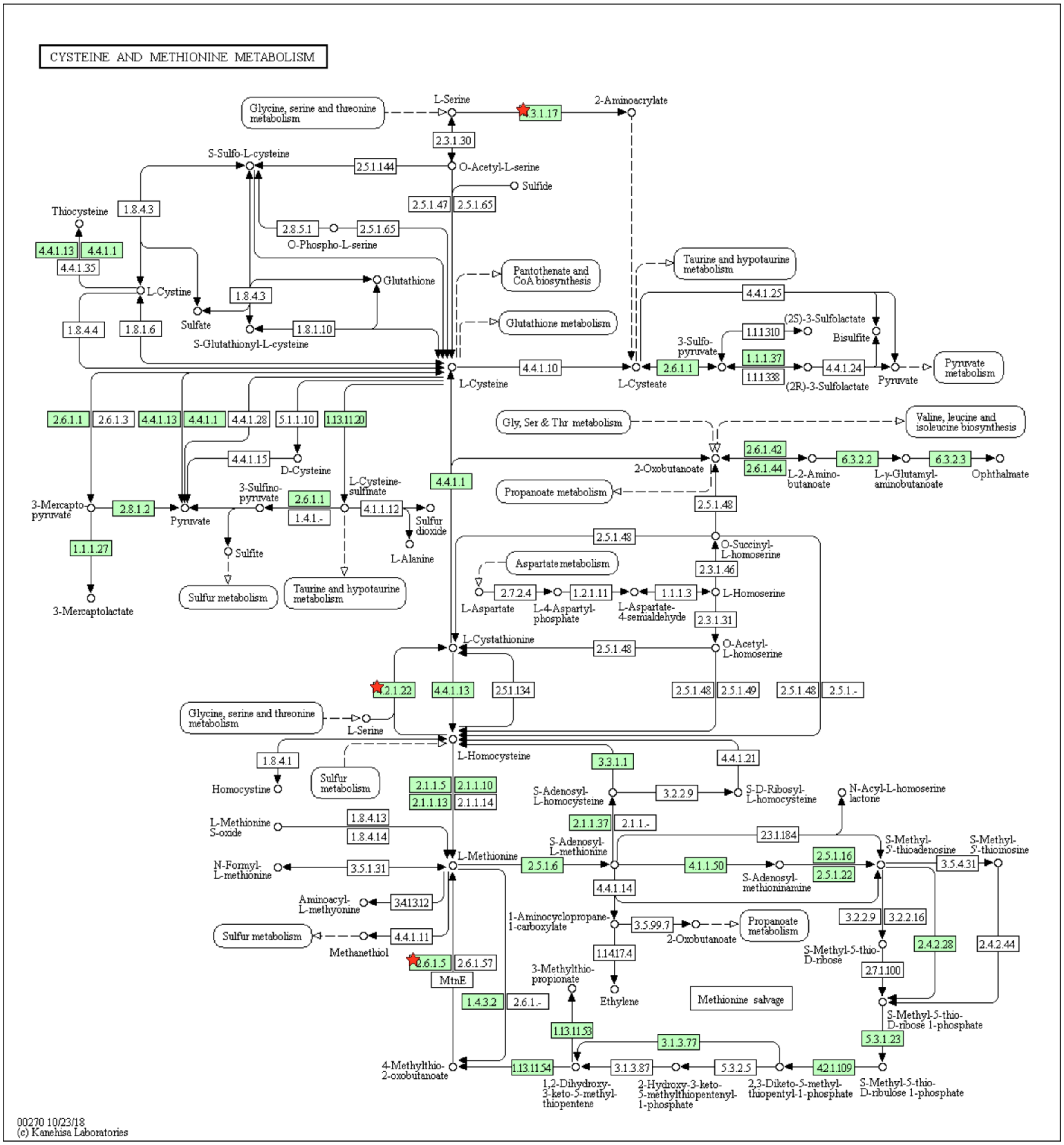

Figure S13. Cysteine and methionine metabolism pathway. Our uploaded genes are marked by red stars. The red genes are labeled by KEGG, and they are in our results.

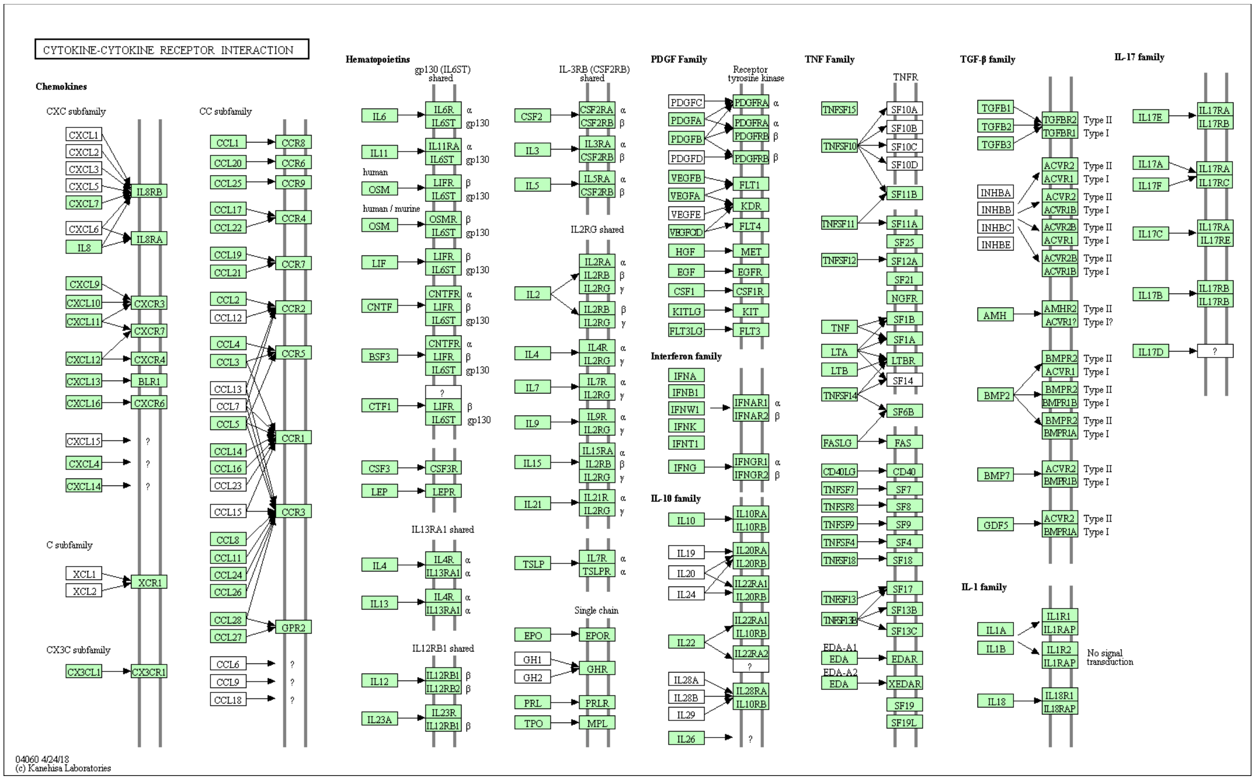

Figure S14. Cytokine-cytokine receptor interaction pathway. Our uploaded genes in this pathway are in the list of gene, but they are not labeled in the map, and they are C-C motif chemokine receptor 5 (CCR5), CD40 molecule (CD40), MET proto-oncogene, receptor tyrosine kinase (MET), epidermal growth factor (EGF), Erythropoietin (EPO), tumor necrosis factor (TNF).

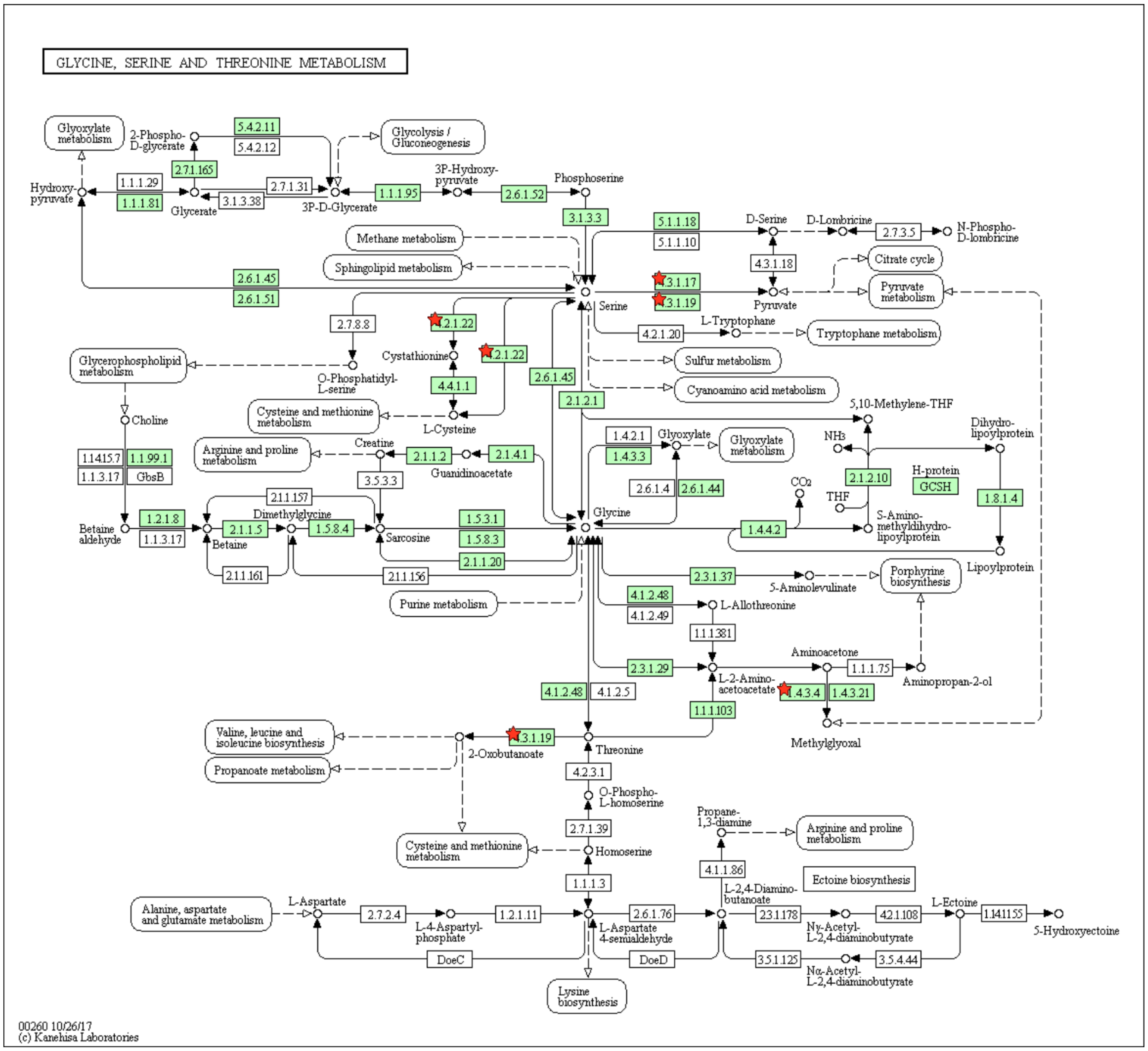

Figure S15. Glycine, serine and threonine metabolism pathway. Our uploaded genes are marked by red stars. The red genes are labeled by KEGG, and they are in our results.

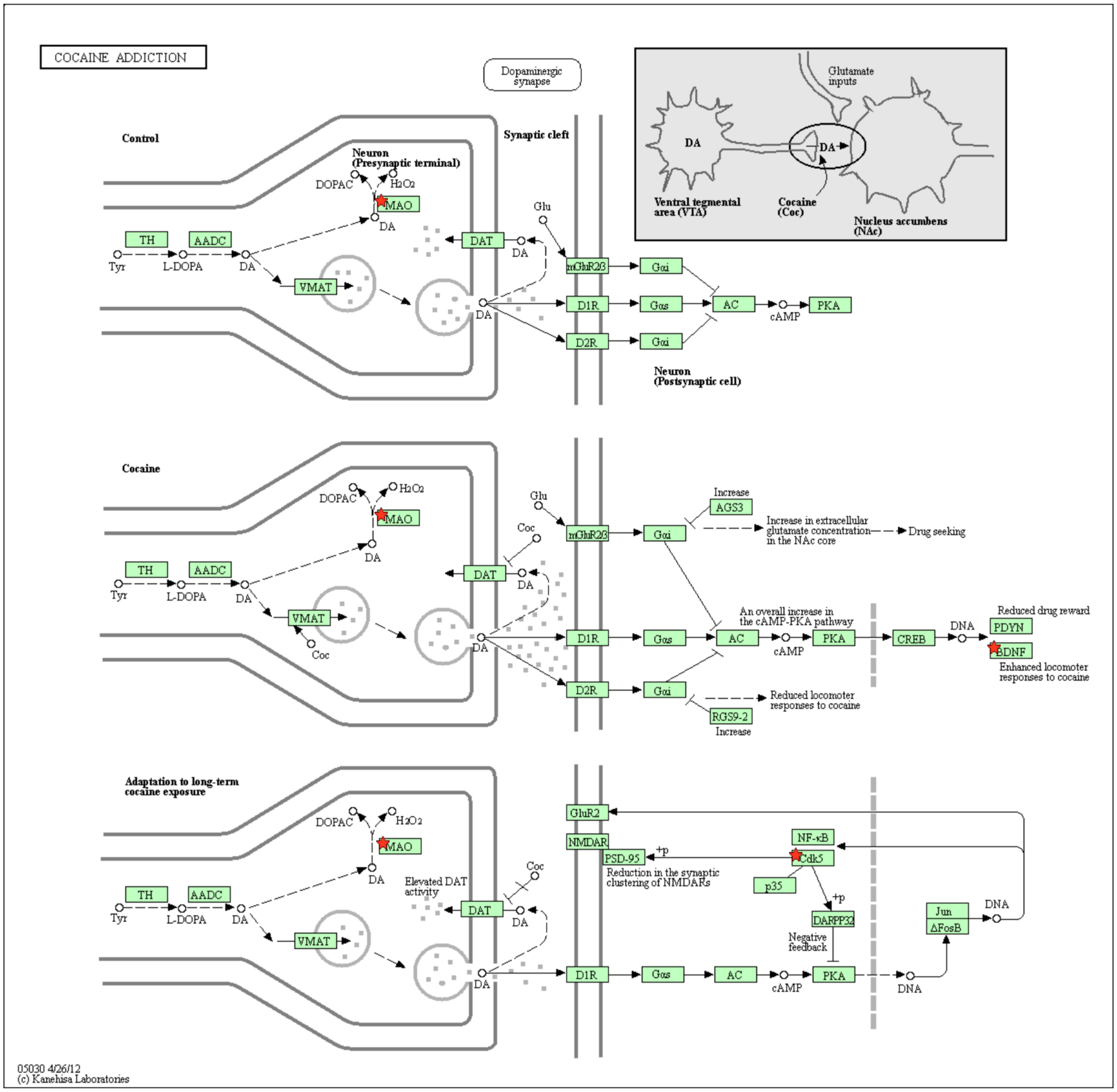

Figure S16. Cocaine addiction pathway. Our uploaded genes are marked by red stars. The red genes are labeled by KEGG, and they are in our results.

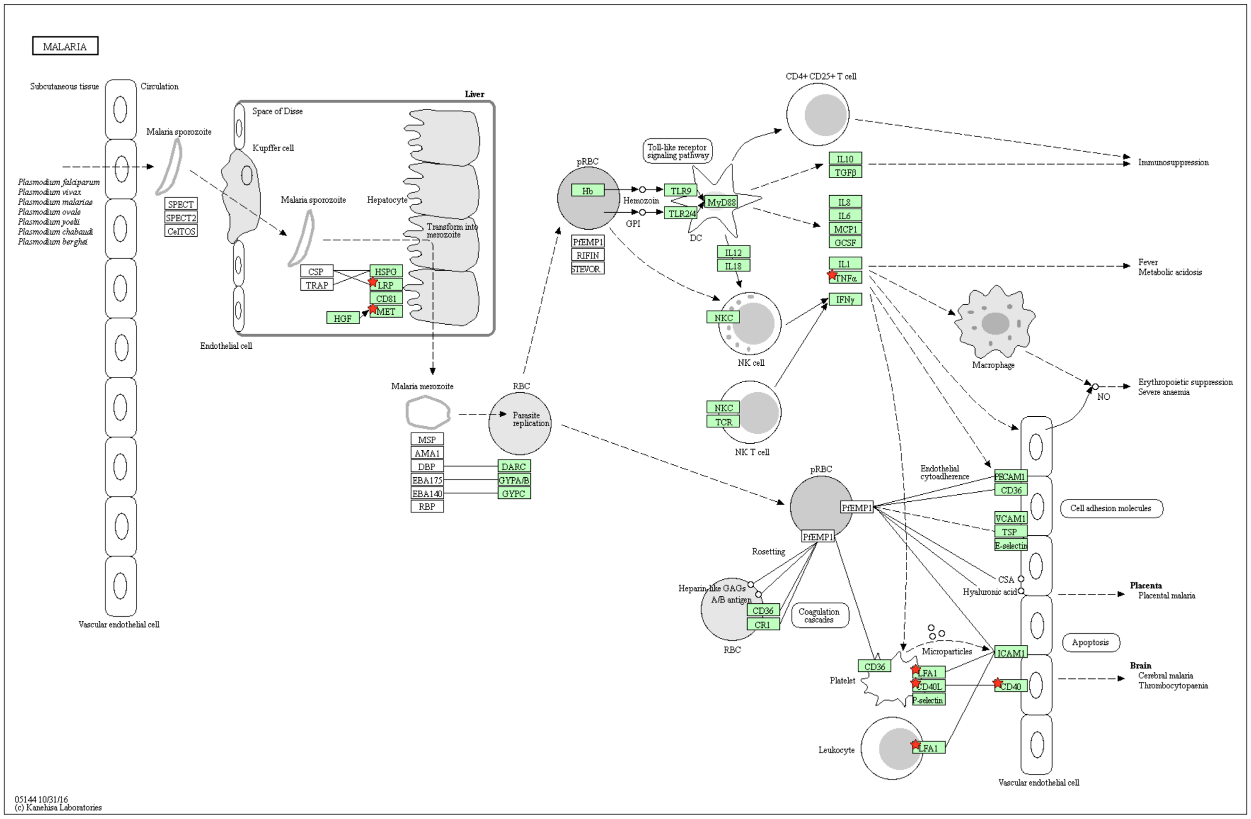

Figure S17. Malaria pathway. Our uploaded genes are marked by red stars. The red genes are labeled by KEGG, and they are in our results.

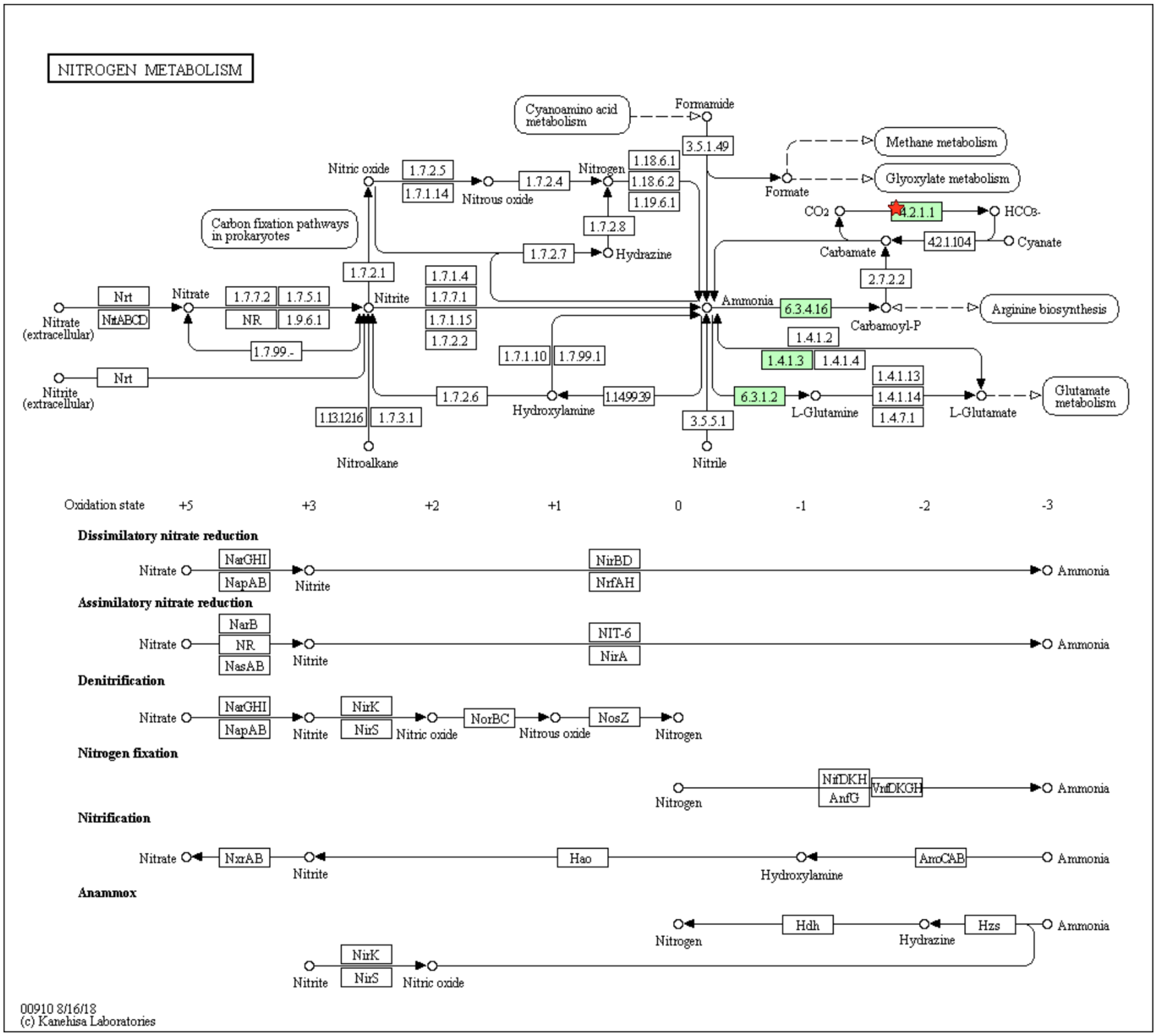

Figure S18. Nitrogen metabolism pathway. Our uploaded genes are marked by red stars. The red genes are labeled by KEGG, and they are in our results.

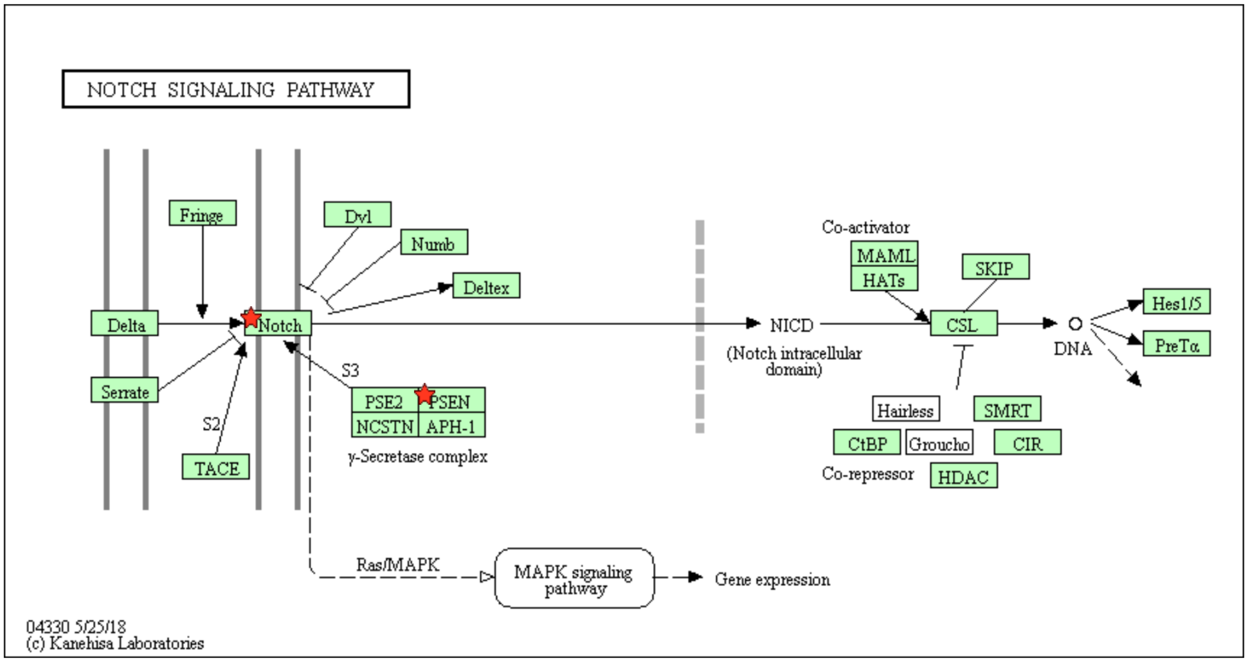

Figure S19. Notch signaling pathway. Our uploaded genes are marked by red stars. The red genes are labeled by KEGG, and they are in our results.

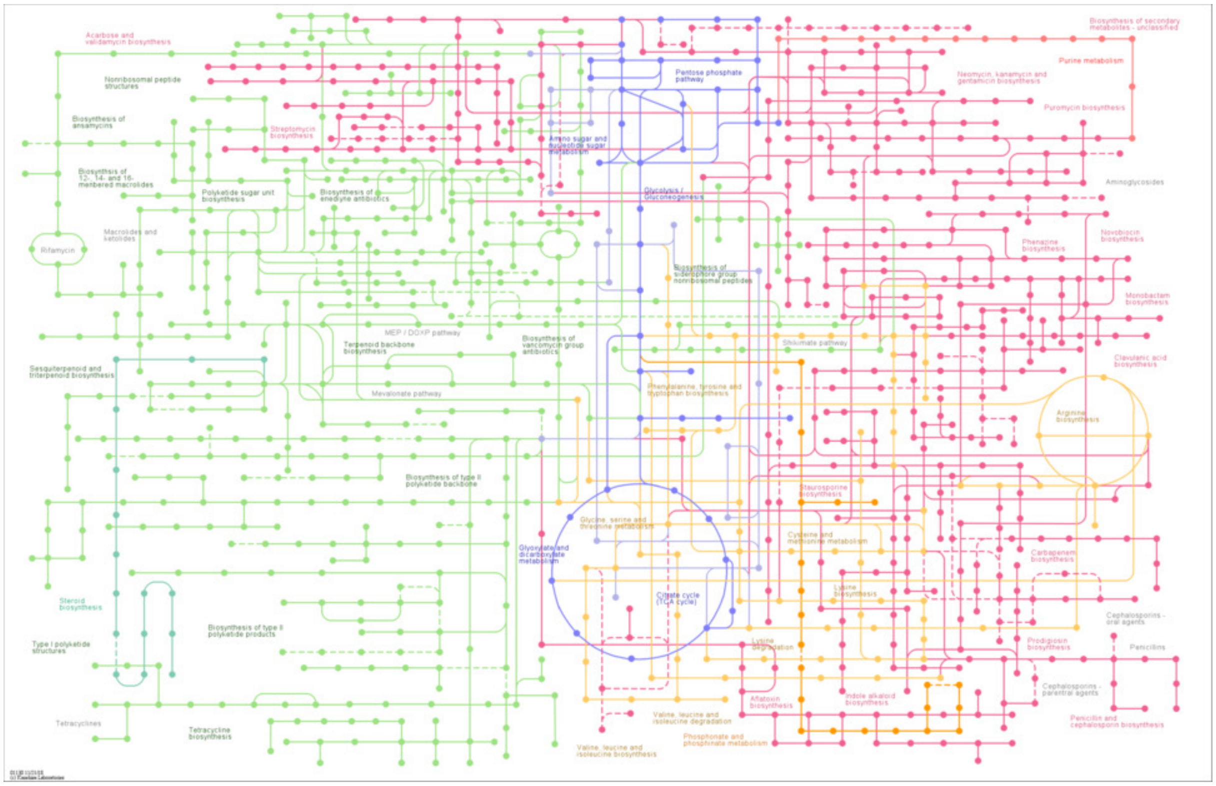

Figure S20. Biosynthesis of antibiotics pathway. The pathway of our uploaded genes cannot be download, so this pathway is the total pathway of Biosynthesis of antibiotics in KEGG database. Our uploaded genes in this pathway are in the list of gene, but they are not labeled in the map, and they are argininosuccinate lyase (ASL), catalase (CAT), citrate synthase (CS), cystathionine-beta-synthase (CBS), serine dehydratase (SDS), tyrosine aminotransferase (TAT).

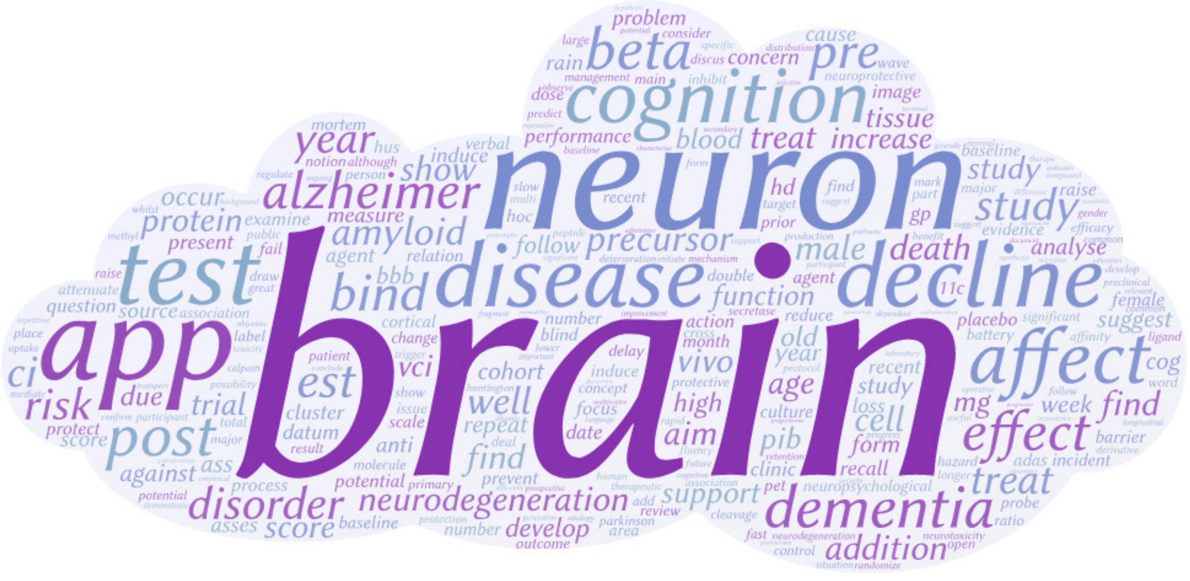

Figure S21. Key topics of AD in 2007. Different size of words displays the word's weight. The higher the word weight is, the bigger the word is.

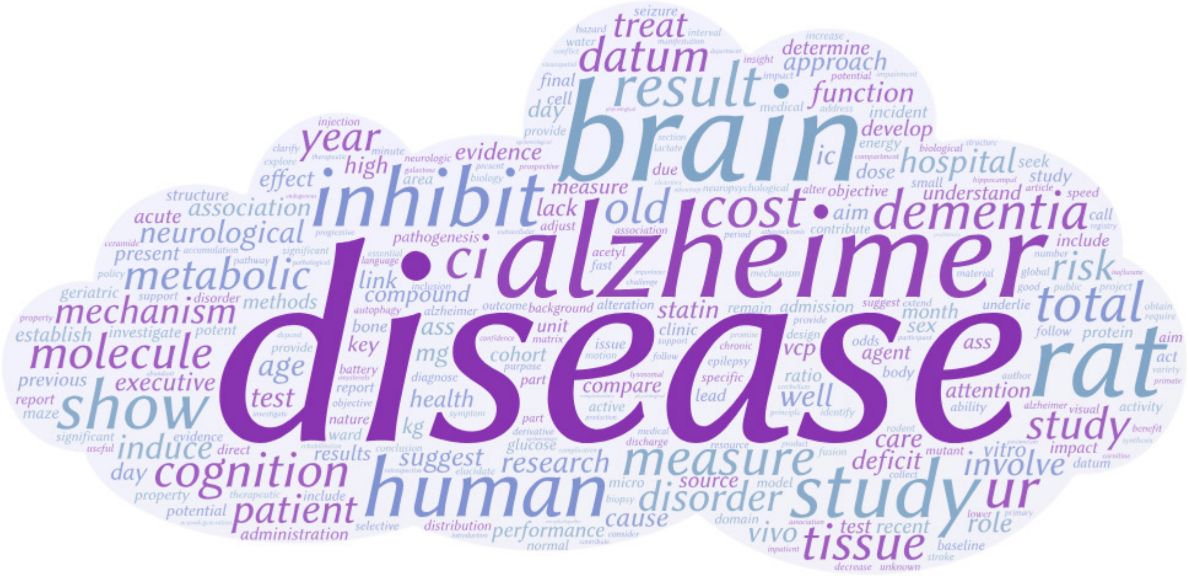

Figure S22. Key topics of AD in 2008. Different size of words displays the word's weight. The higher the word weight is, the bigger the word is.

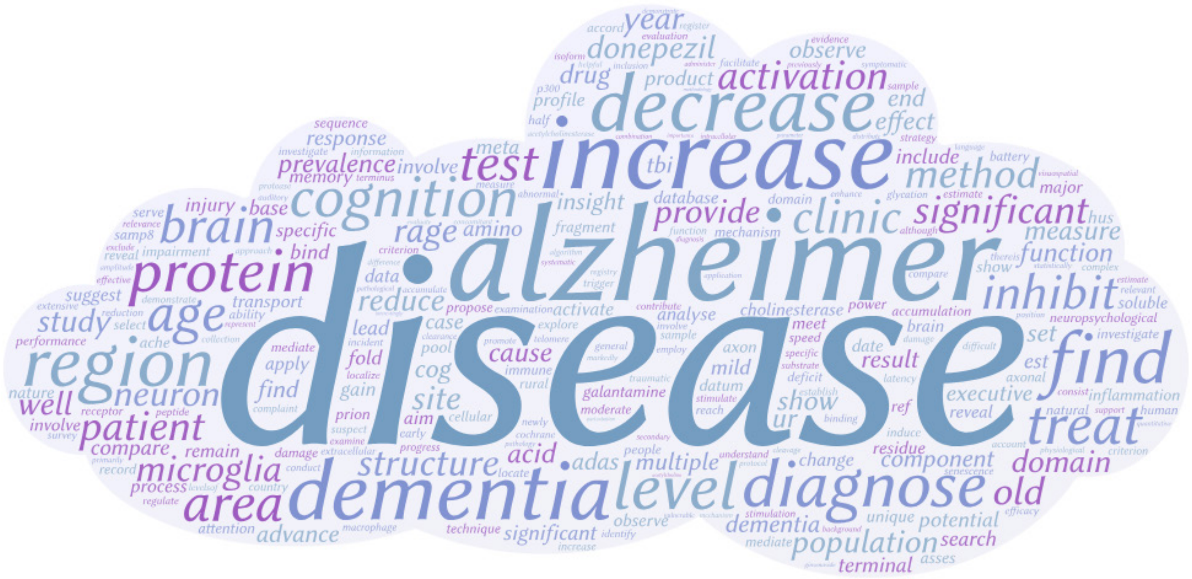

Figure S23. Key topics of AD in 2009. Different size of words displays the word's weight. The higher the word weight is, the bigger the word is.

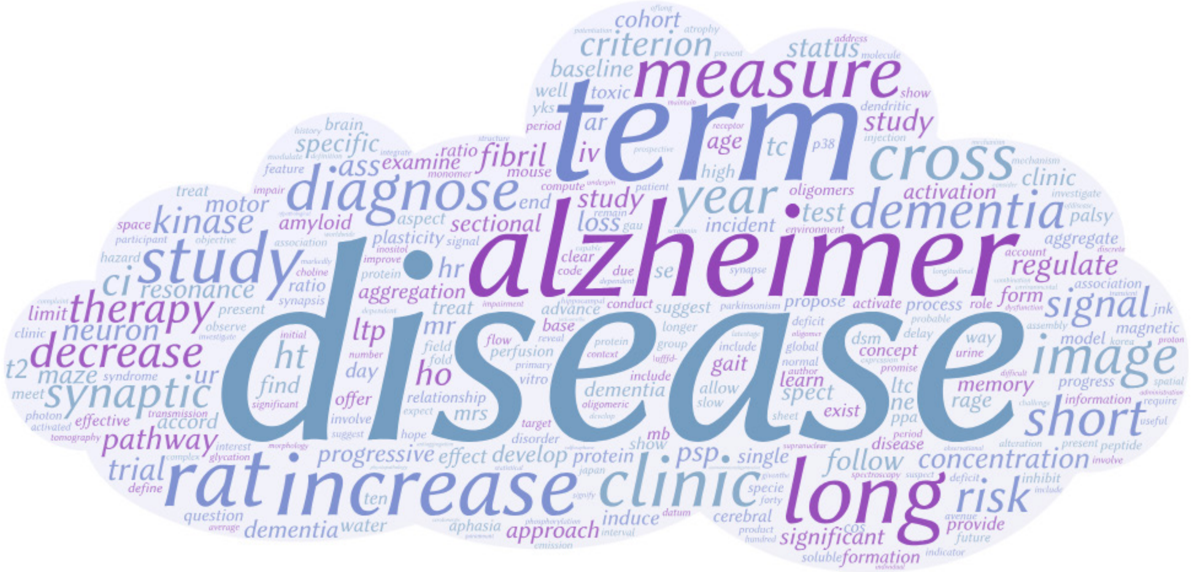

Figure S24. Key topics of AD in 2010. Different size of words displays the word's weight. The higher the word weight is, the bigger the word is.

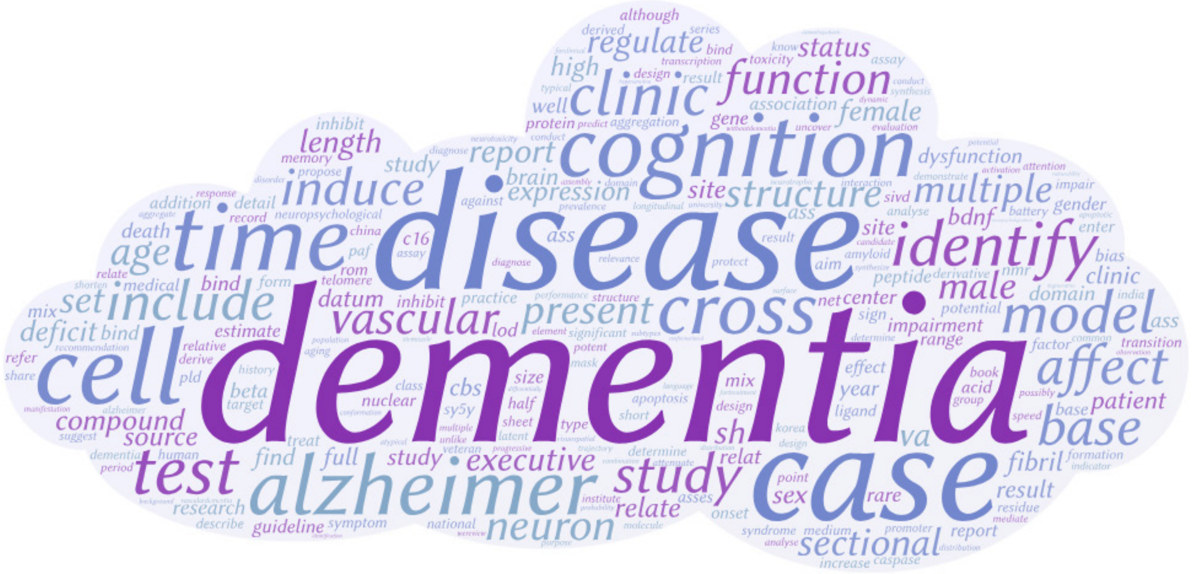

Figure S25. Key topics of AD in 2011. Different size of words displays the word's weight. The higher the word weight is, the bigger the word is.

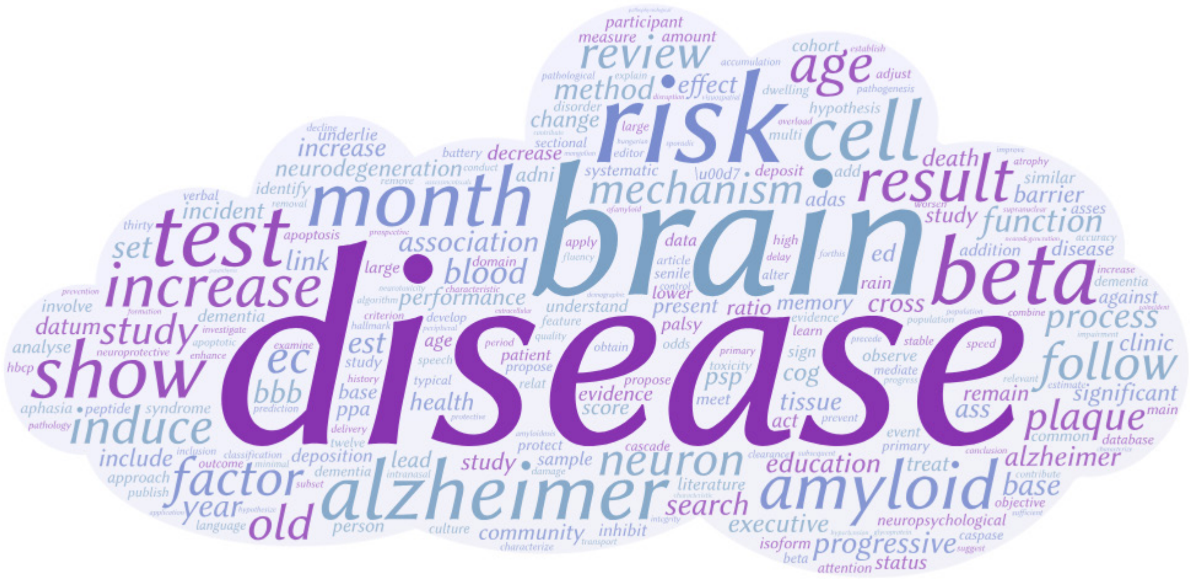

Figure S26. Key topics of AD in 2012. Different size of words displays the word's weight. The higher the word weight is, the bigger the word is.

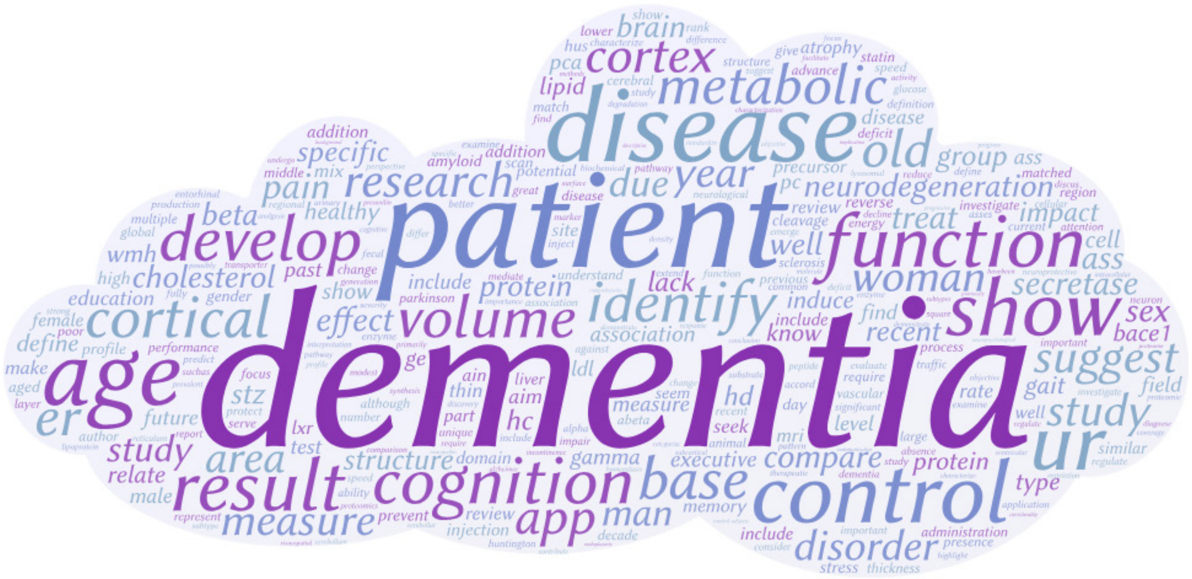

Figure S27. Key topics of AD in 2013. Different size of words displays the word's weight. The higher the word weight is, the bigger the word is.

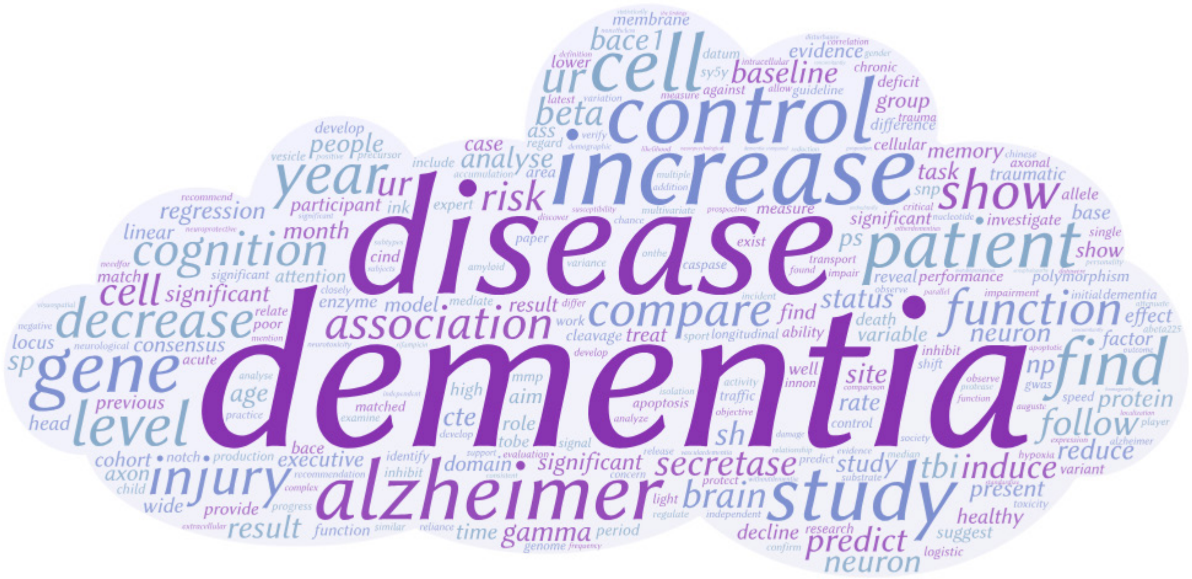

Figure S28. Key topics of AD in 2014. Different size of words displays the word's weight. The higher the word weight is, the bigger the word is.

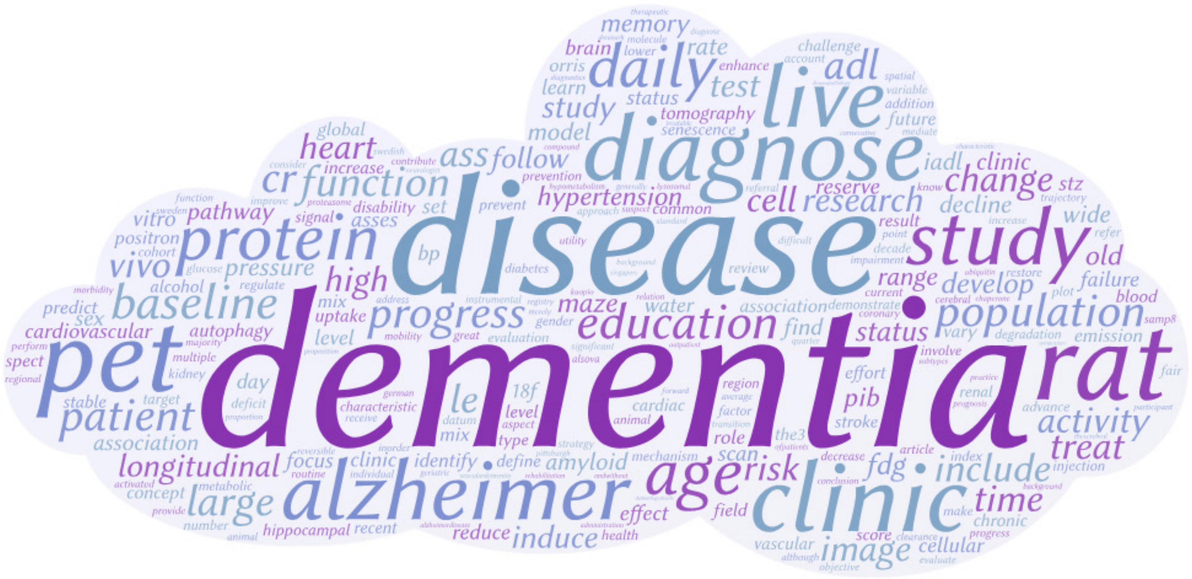

Figure S29. Key topics of AD in 2015. Different size of words displays the word's weight. The higher the word weight is, the bigger the word is.
